# c‐FOS is an integral component of the IKZF1 transactivator complex and mediates lenalidomide resistance in multiple myeloma

**DOI:** 10.1002/ctm2.1364

**Published:** 2023-08-15

**Authors:** Naoki Osada, Jiro Kikuchi, Hidekatsu Iha, Hiroshi Yasui, Sho Ikeda, Naoto Takahashi, Yusuke Furukawa

**Affiliations:** ^1^ Division of Stem Cell Regulation Center for Molecular Medicine Jichi Medical University Tochigi Japan; ^2^ Division of Pathophysiology The Research Center for GLOBAL and LOCAL Infectious Diseases (RCGLID) Oita University Oita Japan; ^3^ Division of Hematology and Oncology, Department of Internal Medicine St. Marianna University School of Medicine Kanagawa Japan; ^4^ Project Division of Innovative Diagnostics Technology Platform, The Institute of Medical Science The University of Tokyo Tokyo Japan; ^5^ Department of Hematology Nephrology and Rheumatology Akita University Graduate School of Medicine Akita Japan; ^6^ Center for Medical Education Teikyo University of Science Tokyo Japan

**Keywords:** activator protein‐1, drug resistance, IKZF1 complex, immunomodulatory drugs, multiple myeloma

## Abstract

**Background:**

The immunomodulatory drug lenalidomide, which is now widely used for the treatment of multiple myeloma (MM), exerts pharmacological action through the ubiquitin‐dependent degradation of IKZF1 and subsequent down‐regulation of interferon regulatory factor 4 (IRF4), a critical factor for the survival of MM cells. IKZF1 acts principally as a tumour suppressor via transcriptional repression of oncogenes in normal lymphoid lineages. In contrast, IKZF1 activates *IRF4* and other oncogenes in MM cells, suggesting the involvement of unknown co‐factors in switching the IKZF1 complex from a transcriptional repressor to an activator. The transactivating components of the IKZF1 complex might promote lenalidomide resistance by residing on regulatory regions of the *IRF4* gene to maintain its transcription after IKZF1 degradation.

**Methods:**

To identify unknown components of the IKZF1 complex, we analyzed the genome‐wide binding of IKZF1 in MM cells using chromatin immunoprecipitation‐sequencing (ChIP‐seq) and screened for the co‐occupancy of IKZF1 with other DNA‐binding factors on the myeloma genome using the ChIP‐Atlas platform.

**Results:**

We found that c‐FOS, a member of the activator protein‐1 (AP‐1) family, is an integral component of the IKZF1 complex and is primarily responsible for the activator function of the complex in MM cells. The genome‐wide screening revealed the co‐occupancy of c‐FOS with IKZF1 on the regulatory regions of IKZF1‐target genes, including *IRF4* and *SLAMF7*, in MM cells but not normal bone marrow progenitors, pre‐B cells or mature T‐lymphocytes. c‐FOS and IKZF1 bound to the same consensus sequence as the IKZF1 complex through direct protein‐protein interactions. The complex also includes c‐JUN and IKZF3 but not IRF4. Treatment of MM cells with short‐hairpin RNA against *FOS* or a selective AP‐1 inhibitor significantly enhanced the anti‐MM activity of lenalidomide in vitro and in two murine MM models. Furthermore, an AP‐1 inhibitor mitigated the lenalidomide resistance of MM cells.

**Conclusions:**

C‐FOS determines lenalidomide sensitivity and mediates drug resistance in MM cells as a co‐factor of IKZF1 and thus, could be a novel therapeutic target for further improvement of the prognosis of MM patients.

## Graphical abstract

Left panel: In multiple myeloma (MM), IKZF1/3 binds to the canonical IKZF‐binding motif CTTCC in a complex with c‐FOS/c‐JUN to activate the transcription of genes involved in the growth and survival of MM cells such as interferon regulatory factor 4 (*IRF4*) and *SLAMF7*.

Middle panel: Lenalidomide induces ubiquitin‐dependent degradation of IKZF1/IKZF3; however, residual members of the MM‐specific activator complex, c‐FOS/c‐JUN, bind to the activator protein‐1 (AP‐1) consensus sequences, which are present in the vicinity of the IKZF‐binding sites of some genes including *IRF4* and *SLAMF7*, leading to sustained expression of these genes and lenalidomide resistance.

Right panel: A selective AP‐1 inhibitor, T‐5224, binds to the DNA‐binding domain of c‐FOS and mitigates the residual activity of the MM‐specific activator complex, resulting in complete *IRF4* down‐regulation to augment the anti‐MM activity of lenalidomide.

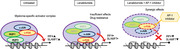

## 1 BACKGROUND

Multiple myeloma (MM) is a blood cancer characterized by the clonal proliferation of transformed plasma cells in the bone marrow.^1^ The treatment outcome of MM patients has been greatly improved by the introduction of proteasome inhibitors as well as thalidomide and its analogues lenalidomide and pomalidomide, which are collectively designated immunomodulatory drugs (IMiDs).^2^ More recently, therapeutic antibodies against CD38 and SLAMF7 have been incorporated into treatment strategies and quickly became an essential part of the current standard of care with IMiDs.^3^ Among these key drugs, lenalidomide is arguably the most important and is continuously used during the course of MM treatment.^4^ Low sensitivity to lenalidomide and the emergence of resistance lead to early treatment failure and late relapse during maintenance, respectively.^5^ Therefore, the identification of the factors determining lenalidomide sensitivity is critical for further improvement of the treatment outcome of MM patients.

IMiDs exert anti‐myeloma action via direct binding to cereblon (CRBN), the substrate receptor of the CRL4 E3 ubiquitin ligase complex.^6^ CRBN‐bound IMiDs recruit IKZF1/Ikaros and IKZF3/Aiolos, members of the IKZF family of transcription factors, for ubiquitination and subsequent proteasome‐dependent degradation.^7^ The degradation of IKZF1 and IKZF3 results in the diminished expression of interferon regulatory factor 4 (IRF4), which is essential for the survival of MM cells,^8^ and subsequent cell death.^6^, ^7^ Given the fundamental role of CRBN in the anti‐myeloma activity of IMiDs, it is reasonable to speculate that its dysfunction underlies cell insensitivity and resistance to IMiDs. In fact, Gooding et al.^9^ reported the association of genetic alterations of the *CRBN* gene, such as point mutations, structural abnormalities, and alternative splicing, with acquired resistance to both lenalidomide and pomalidomide in MM patients. However, the genetic changes in *CRBN* do not explain lenalidomide resistance in all cases, because the abnormalities, excluding exon 10 splicing, are detected in less than 10% of lenalidomide‐refractory cases.^10^, ^11^ Therefore, it is still important to explore additional mechanisms of lenalidomide resistance in MM.

The IKZF family is widely expressed in the hematopoietic system and maintains homeostasis through the transcriptional regulation of a wide variety of genes involved in the proliferation and differentiation of blood cells.^12^ The first member of the family, IKZF1/Ikaros, functions as a tumour suppressor in lymphoid lineages via transcriptional repression of *NOTCH1* in association with the nucleosome remodelling and deacetylase (NuRD) complex and the Polycomb repressive complex 2.^13^, ^14^ In normal lymphoid cells, the repressor function of IKZF1 is dominant over its activator function for the maintenance of lineage fidelity.^15^ In contrast, in MM cells, IKZF1 acts as a transcriptional activator to sustain the expression of *IRF4* and other critical oncogenes.^16^, ^17^ It is tempting to speculate that IKZF1 forms a complex in MM cells that differs from the complex in normal lymphoid cells and that a component of the MM‐specific IKZF1 complex mediates transactivator function. In support of this view, a recent report demonstrates that IKZF1 knockdown does not necessarily suppress the expression of IRF4 in MM cells.^18^ This finding indicates the existence of an unknown factor that could work independently of and/or in cooperation with IKZF1 to mediate oncogenic transcription in MM cells and could promote IMiD resistance by maintaining *IRF4* transcription after IKZF1 degradation.

In this study, we attempted to identify the factor that changes the IKZF1 complex from a transcriptional repressor to an activator in MM cells using bioinformatic approaches. We found that c‐FOS, a member of the activator protein‐1 (AP‐1) family of transcription factors,^19^, ^20^ is an integral component of the IKZF1 complex and is primarily responsible for the activator function of the complex in MM cells. The expression levels of c‐FOS appear to determine the sensitivity of MM cells to lenalidomide; c‐FOS mediates lenalidomide resistance via a compensatory increase in the amounts of c‐FOS bound on promoter regions of IKZF1‐target genes after lenalidomide treatment. c‐FOS may be a novel target for augmenting lenalidomide efficacy and overcoming lenalidomide resistance in MM cells.

## 2 MATERIALS AND METHODS

### 2.1 Drugs

We purchased lenalidomide and T‐5224 from Selleck Chemicals and other reagents from Sigma‐Aldrich unless otherwise stated. Drugs were dissolved in dimethyl sulfoxide (DMSO) and used at a final dilution of 1/1000 to keep the final concentrations of DMSO < 0.1% to prevent alterations of drug effects or cell growth.

### 2.2 Cells and cell culture

We obtained the human myeloma cell lines MM.1S, KMM.1, KMS12‐BM, KMS21, KMS26, KMS28‐BM, KMS34, SK‐MM‐2, NCI‐H929 and RPMI8226 from the Health Science Research Resources Bank, where the cell line authenticity and absence of Mycoplasma infection are routinely checked by DNA fingerprinting and PCR. Lenalidomide‐resistant sublines of NCI‐H929 designated NCI‐H929LR, were established by continuous exposure of parent cells to increasing concentrations of lenalidomide. Primary bone marrow cells were isolated from MM patients at the time of the diagnostic procedure and used after the positive selection of MM cells using CD138 MicroBeads and MACS separation columns (Miltenyi Biotech). We obtained written informed consent from all patients in accordance with the Declaration of Helsinki. The protocol was approved by the Institutional Review Boards of Jichi Medical University and Akita University Graduate School of Medicine.

### 2.3 Construction and production of lentiviral expression vectors

We used the lentiviral short‐hairpin RNA/short‐interfering RNA (shRNA/siRNA) expression vector pLL3.7 for c‐FOS knockdown experiments. Oligonucleotides containing siRNA target sequences are shown in Table S1. Scrambled sequences were used as controls. These vectors were co‐transfected into 293FT cells with packaging plasmids (Thermo Fisher Scientific) to produce infective lentiviruses in culture supernatants. Lentiviruses were then added to cell suspensions in the presence of 8 μg/ml polybrene and transduced for 24  h.

### 2.4 Quantitative real‐time reverse transcription‐PCR

Total cellular RNA was isolated using an RNeasy Kit (Qiagen), reverse‐transcribed into complementary DNA using ReverTra Ace and oligo(dT) primers (Toyobo), and subjected to quantitative real‐time reverse transcription‐PCR using Expression Assays (Hs99999140 for *FOS*, Hs00180031 for *IRF4*, Hs00904275 for *SLAMF7*, Hs00936295 for *BSG*, Hs00152927 for *CD48* and Hs01922876 for *GAPDH*) and TaqMan Fast Universal PCR Master Mix.

### 2.5 Immunoprecipitation and immunoblotting

We prepared nuclear and cytoplasmic fractions using nuclear and cytoplasmic extraction reagents (Cayman Chemical).^21^ For immunoprecipitation, we incubated the primary antibody with protein A magnetic beads (Thermo Fisher Scientific) at 4°C for 24 h, added cell lysates to the antibody‐bound beads in solution after discarding the supernatants, and rotated the samples for 30 min. After washing, immunoprecipitates were eluted and subjected to SDS‐PAGE, followed by immunoblotting using antibodies against IKZF1 (#14859), IKZF3 (#15103), c‐FOS (#2250), FOSB (#2251), c‐JUN (#9165) (Cell Signaling Technology, Beverly, MA), IRF4 (sc‐48338), GAPDH (sc‐47724) (Santa Cruz Biotechnology), normal rabbit IgG (PM035) and FLAG tag (PM020) (MBL).

### 2.6 Chromatin immunoprecipitation assays

We used ChIP‐IT Express Enzymatic Kit (Active Motif, Carlsbad, CA) to perform ChIP assays.^22^ In brief, we fixed cells in 1% formaldehyde at room temperature for 10 min and isolated chromatin fractions using enzymatic shearing. After centrifugation, the supernatants were incubated with ChIP‐grade antibodies against IKZF1/Ikaros (Active Motif 2A9) and c‐FOS (Active Motif 2C5) or isotype‐matched controls in the presence of protein G magnetic beads at 4°C overnight. We purified DNA fragments from the mixture and carried out quantitative PCR using Power SYBR Green Master Mix (Thermo Fisher Scientific) and the primers listed in Table S2. The fold enrichment of target genes between ChIP‐DNA and input‐DNA was determined by the 2^−∆∆Ct^ method. We outsourced the ChIP‐seq analyses to Active Motif and deposited the data and protocols in the MINSEQE‐compliant GEO database under accession numbers GSE194381 and GSE194387.

### 2.7 Protein‐DNA binding assays

We examined whether IKZF1 and c‐FOS formed a stable complex on DNA using an EpiQuik General Protein‐DNA Binding Assay Kit (EpiGentek) as reported previously.^23^ In brief, we incubated nuclear extracts with a biotinylated double‐stranded oligonucleotide harbouring an IKZF‐binding sequence, TCAGCTTTTGGGAATGTATTCCCTGTCA,^24^ and captured the resultant IKZF‐DNA complex on the bottom of streptavidin‐coated plates. After vigorous washing, the residual complex was detected by sandwich immunoassay using an anti‐IKZF1 antibody (Thermo Fisher Scientific 4E9) and a horseradish peroxidase‐conjugated secondary antibody. The DNA‐binding activity level was calculated according to the manufacturer's instructions. For antibody perturbation, we included specific antibodies against c‐FOS (Cell Signaling Technology #2250) and IKZF1 (Cell Signaling Technology #14859) during the incubation of nuclear extracts with the oligonucleotide probe.

### 2.8 Murine MM models

Luciferase‐expressing sublines of MM.1S cells were suspended in 100 μl of RPMI‐1640 medium and subcutaneously inoculated into the right thigh of male nonobese diabetic/severe combined immunodeficiency (NOD/SCID) mice (Charles River Laboratories). Drugs were administered intraperitoneally in 200 μl of solution containing 5% DMSO (vol/vol) in sterile phosphate‐buffered saline. The control group received only a vehicle following the same treatment schedule as the experimental group. The tumour burden was monitored by measuring tumour‐derived luciferase activity with a noninvasive bioimaging system. In short, tumour‐bearing mice were intraperitoneally injected with 1.5 mg of the luciferase substrate *D*‐luciferin after being anaesthetized with isoflurane. Photons transmitted through the body were collected for a specified length of time and analyzed using the IVIS‐CT Imaging System with Living Image software (Xenogen). The quantitative data are expressed as photon units (photons/s).^25^, ^26^, ^27^ We also used an orthotopic MM model, in which luciferase‐expressing KMS26 cells were engrafted into the bone marrow of NOD/SCID mice via tail‐vein injection. All animal studies were approved by the Institutional Animal Ethics Committee and performed in accordance with the Guide for the Care and Use of Laboratory Animals formulated by the National Academy of Sciences.

### 2.9 Histopathological examination of inoculated tumours

Subcutaneous tumours were resected from euthanized mice, weighed, and embedded in the Tissue‐Tek O.C.T. compound (Sakura Finetek) after formalin fixation. We prepared 5‐μm‐thick sections and subjected them to hematoxylin‐eosin staining or immunohistochemical staining with mouse anti‐CD138 (Thermo Fisher Scientific DL‐101) and rabbit anti‐IRF4 (Cell Signaling Technology #15106) antibodies as primary antibodies and Alexa Fluor 488‐conjugated anti‐rabbit IgG and Alexa Fluor 594‐conjugated anti‐mouse IgG (Thermo Fisher Scientific) as secondary antibodies. Nuclei were counterstained with DAPI (Cell Signaling Technology #8961). The stained samples were examined under a BZ‐X fluorescence microscope (Keyence, Osaka, Japan).

### 2.10 Statistics

We used KaleidaGraph software (Synergy Software) to perform one‐way ANOVA with the Student–Newman–Keuls multiple comparison test and Student's *t*‐test to determine statistical significance. Kaplan‐Meier survival curves were generated and analyzed by log‐rank test using GraphPad Prism software (GraphPad Software). *p‐*Values less than .05 were considered significant.

## 3 RESULTS

### 3.1 Co‐binding of IKZF1 and c‐FOS at transcription start sites of IKZF1‐target genes in MM cells

**FIGURE 1:**
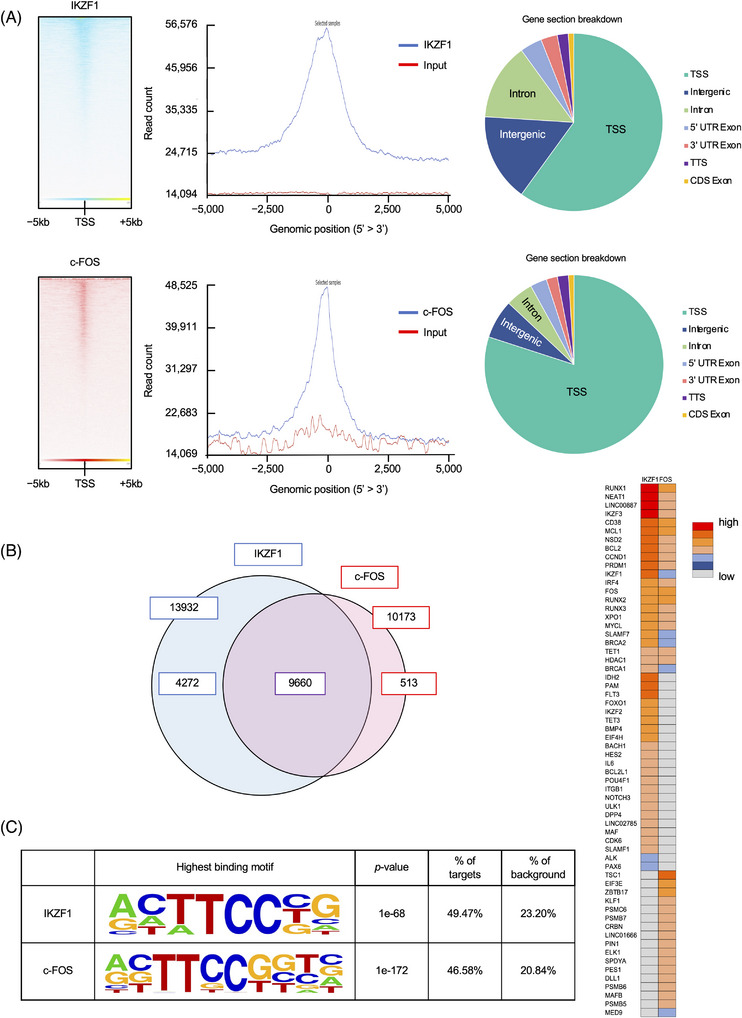
Global analyses of IKZF1 and c‐FOS binding to the myeloma genome. (A) Upper panel: Average plot (middle) and heatmap (left) of IKZF1 chromatin immunoprecipitation (ChIP)‐seq reads over all transcription start sites (TSSs) ± 5000 bp. The pie chart shows the gene section breakdown (right). Lower panel: Average plot (middle) and heatmap (left) of c‐FOS ChIP‐seq reads over all TSSs ± 5000 bp. The pie chart shows the gene section breakdown (right). Genes (rows) were ordered in the same way in heatmaps. (B) Left panel: Overlap of IKZF1‐ (*n* = 13 932) and c‐FOS‐binding (*n* = 10 173) sites in MM.1S cells. Right panel: Status of IKZF1 and c‐FOS binding at promoter/enhancer regions of representative genes based on ChIP‐seq data. (C) Nucleotide sequences of IKZF1‐ and c‐FOS‐binding sites deduced from ChIP‐seq analyses of MM.1S cells. The ChIP‐seq data were analyzed using a Partek Flow genomic analysis software v10.0 (Partek Inc.).

**FIGURE 2:**
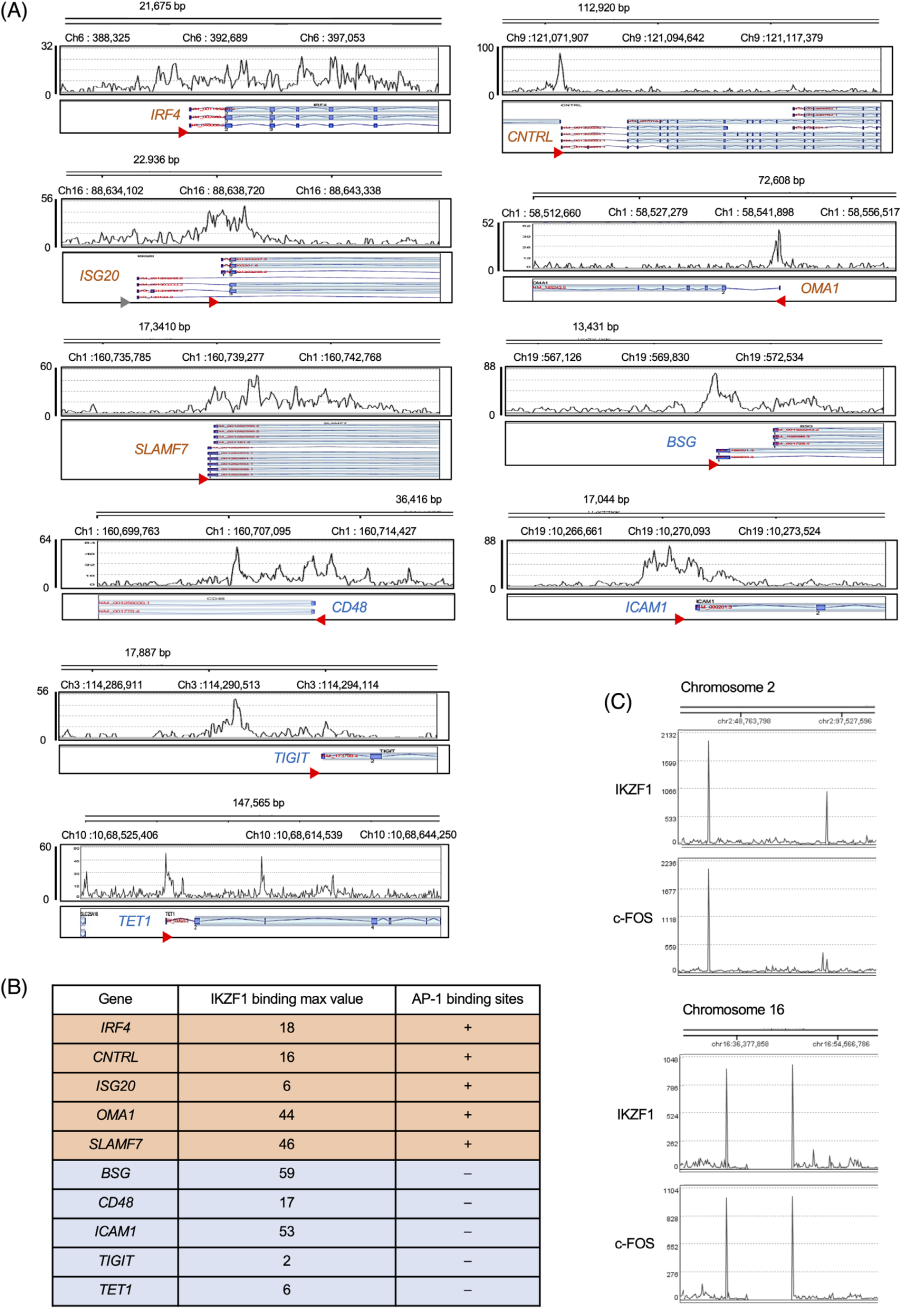
c‐FOS‐binding sites are present in nearly one‐half of IKZF1‐target genes in multiple myeloma (MM). (A) Using chromatin immunoprecipitation (ChIP)‐seq data of MM.1S cells, we visualized IKZF1 binding near transcription start sites (TSSs) (red triangles) of the indicated genes in the UCSC genome browser. The genes possessing the activator protein‐1 (AP‐1)‐binding motif based on a TRANSFAC search (https://genexplain.com/transfac/) are marked in red, and those without the conventional AP‐1‐binding motif are marked in blue. (B) The peak values of IKZF1 binding and the presence of AP‐1‐binding sites in selected genes. (C) An example of the co‐occupancy of IKZF1 and c‐FOS in MM.1S cells.

**FIGURE 3:**
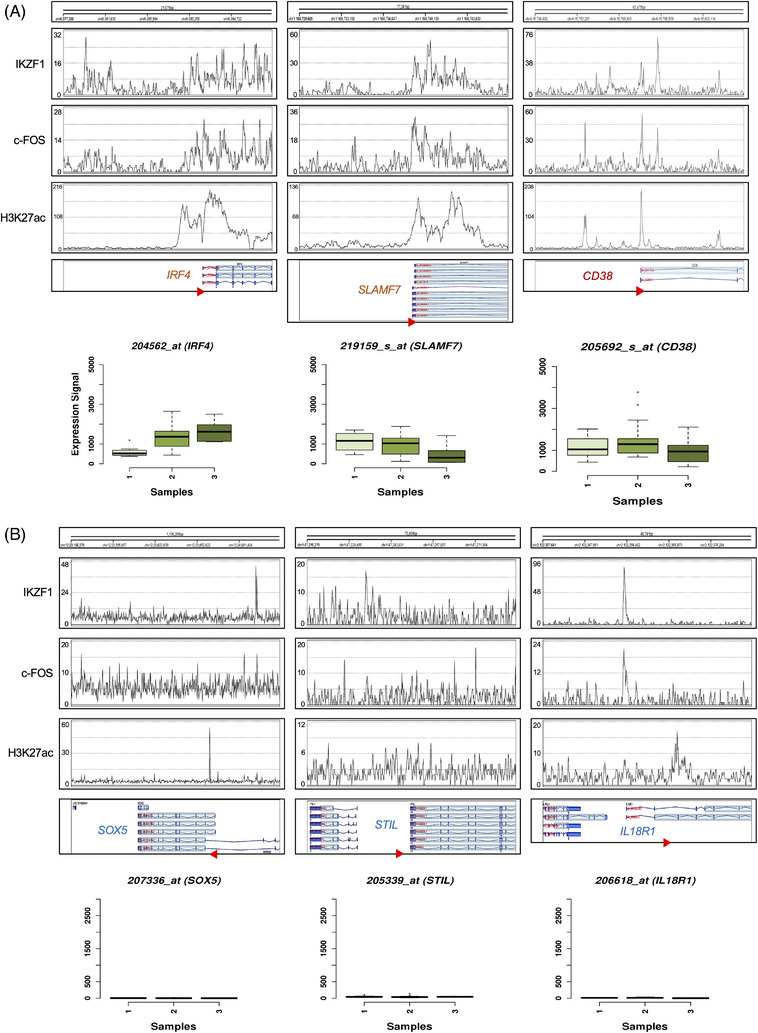
Co‐occupancy of IKZF1 and c‐FOS at promoter/enhancer regions of actively transcribed genes in multiple myeloma (MM). Upper panel: chromatin immunoprecipitation (ChIP)‐seq data of IKZF1 and c‐FOS binding in MM.1S cells were aligned with acetylated histone H3K27 marks in the UCSC genome browser. The transcription start site (TSS) of each gene is shown with red triangles. Lower panel: Gene expression was assayed using Affymetrix U133 plus 2.0 microarrays. The data are unpaired GCRMA‐normalized expression signals for each gene in CD138‐positive cells from patients with (1) monoclonal gammopathy of undetermined significance, (2) newly‐diagnosed MM and (3) plasma cell leukaemia (*n* = 8 each).^53^ (A) The results of three representative genes that are highly expressed in plasma cell disorders. (B) The results of three representative genes not expressed in plasma cell disorders.

To identify the unknown components of the MM‐specific IKZF1 complex in situ, we comprehensively analyzed the binding of IKZF1 to the genome of the human MM cell line MM.1S using chromatin immunoprecipitation with high‐throughput DNA sequencing (ChIP‐seq). The genome‐wide screening of IKZF1 binding detected 13,932 peaks, ∼60% of which were located in the vicinity of transcription start sites (TSSs) in MM.1S cells (Figure 1). We confirmed the binding of IKZF1 at the TSSs of well‐known targets, such as *IRF4*, *SLAMF7* and *BSG*,^7^, ^16^, ^28^, ^29^ and identified novel candidate IKZF1‐regulated genes, such as *CNTRL*, *ISG20*, *OMA1*, *CD48*, *ICAM1*, *TIGIT* and *TET1*, on the basis of enhancer/promoter binding (Figure 2A). Next, we incorporated the results of ChIP‐seq analyses of IKZF1 binding into the ChIP‐Atlas platform^30^, ^31^ and investigated the co‐occupancy of IKZF1 with other DNA‐binding factors on the genome of MM cells. ChIP‐Atlas analyses revealed that the AP‐1 binding motif was present in nearly half of IKZF1‐target genes and that c‐FOS, a member of the AP‐1 family, was actually co‐localized with IKZF1 at histone H3K27‐acetylated enhancer/promoter regions of these genes in MM cells (Figure S1 and Figure 2B). Co‐localization of IKZF1 and c‐FOS was not evident on the *IRF4* or *SLAMF7* gene in normal bone marrow progenitors, pre‐B cells or CD4‐positive T‐lymphocytes, although c‐FOS alone bound to the *IRF4* promoter in pre‐B cells and IKZF1 alone bound to the *SLAMF7* promoter in helper T‐cells, probably reflecting their involvement in active transcription of respective genes in each cell type (Figure S2). To validate the co‐occupancy of IKZF1 and c‐FOS on the myeloma genome, we carried out ChIP‐seq analyses of global c‐FOS binding in MM.1S cells and superimposed the two images on the UCSC genome browser. The genome‐wide screening of c‐FOS binding detected 10 173 peaks, ∼80% of which were located in the vicinity of TSSs in MM.1S cells (Figure 1). Whole‐chromosome images generated by Strand NGS software revealed a significant overlap of the peaks representing IKZF1 and c‐FOS binding to the myeloma genome (Figure 1A, left panel, and Figure 2C). In detail, IKZF1 was co‐occupied in 95% (9660/10 173) of c‐FOS‐binding sites, and conversely, c‐FOS localization was observed in 69% (9660/13 932) of IKZF1‐binding sites in MM cells (Figure 1B, left panel). Binding motif analyses revealed that the consensus sequence 5′‐ACTTCC(C/G)G‐3′, which contains the GGAA core of the IKZF‐binding motif in reverse orientation,^13^ were significantly enriched and detected in 49.47% of IKZF1‐binding sites and 46.58% of c‐FOS‐binding sites in MM.1S cells (Figure 1C). This indicates that the two factors bind to the same sequence in approximately 50% of target genes. Co‐occupancy was detected in canonical IKZF1 targets, such as *IRF4* and *SLAMF7*, as well as in genes closely related to MM biology including *CD38, MCL1, NSD2/MMSET, CCND1, PRDM1, IKZF1* and *IKZF3* (Figure 1B, right panel). Moreover, the two factors accumulated in the regions where histone H3K27 was heavily acetylated in genes expressed at high levels in MM cells (Figure 3A and Figure S3A), whereas co‐occupancy was not observed or was not co‐localized with histone H3K27 acetylation in genes underexpressed in MM cells (Figure 3B and Figure S3B). These results strongly suggest that c‐FOS is a co‐factor that works in concert with IKZF1 in the transcriptional activation of genes related to MM biology, in addition to *IRF4*.

### 3.2 c‐FOS is actually involved in the transcriptional regulation of IKZF1‐target genes in MM cells

**FIGURE 4:**
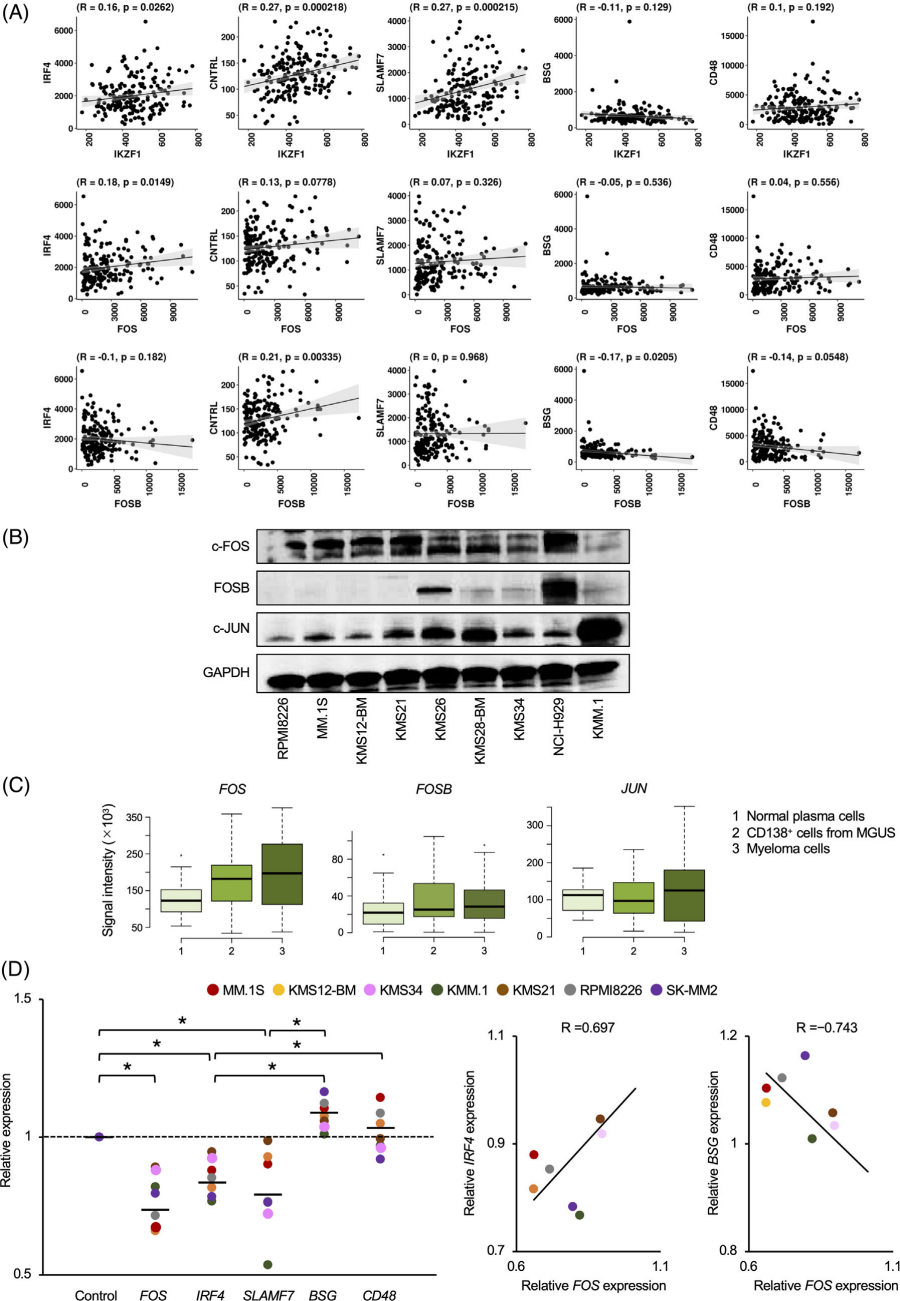
Biological functions of activator protein‐1 (AP‐1) family proteins in multiple myeloma (MM). (A) We determined the correlation of expression levels between IKZF1‐target genes (y‐axis) and the indicated genes (x‐axis) using the GenomicScape tool (http://www.genomicscape.com).^32^ The data were extracted from DNA microarray analyses of gene expression in newly‐diagnosed MM patients.^53^ (B) Cell lysates were prepared from the indicated MM cell lines and analyzed by immunoblotting using antibodies against c‐FOS, FOSB, c‐JUN and GAPDH (loading control). (C) We examined the expression of *FOS*, *FOSB* and *JUN* transcripts in normal plasma cells (*n* = 22), CD138‐positive cells derived from patients with monoclonal gammopathy of undetermined significance (MGUS; *n* = 44) and MM cells from newly‐diagnosed patients (*n* = 12). The data were extracted from DNA microarray analyses deposited in the GenomicScape database by the Arkansas group.^53^ (D) Left panel: We transduced the indicated MM cell lines with shRNA against *FOS* or scrambled sequences (sh‐control) and evaluated the expression of candidate IKZF1‐target genes (*IRF4*, *SLAMF7*, *BSG* and *CD48*) along with *GAPDH* (Control) and *FOS* (for the evaluation of knockdown efficiency) using quantitative real‐time reverse transcription‐PCR after 24 h. The data were quantified by the 2^–∆∆Ct^ method using *GAPDH* as a reference and are shown as the fold changes against the values of sh‐control‐transfected cells. *p < .05 by one‐way ANOVA with Student–Newman–Keuls multiple comparison tests. Right panel: Correlation between the expression levels of *FOS* mRNA and those of *IRF4* or *CD48* mRNA in MM cell lines.

Having demonstrated that c‐FOS is bound to histone H3K27‐acetylated regulatory regions of IKZF1‐target genes, we investigated whether c‐FOS is actually involved in the transactivation of these genes in MM cells. First, we analyzed the GenomicScape database of gene expression in primary MM cells from newly‐diagnosed patients^32^ and found a weak (R = 0.18) but significant (*p* = 0.0149) correlation between the expression levels of *FOS* and *IRF4* transcripts at levels equivalent to the correlation between *IKZF1* and *IRF4* (R = 0.16, *p* = 0.0262) (Figure 4A, upper and middle panels). On the other hand, *FOSB*, another member of the FOS family, was not correlated with the expression of bona fide IKZF1 targets in MM cells (Figure 4A, lowermost panel). To gain a better understanding of the physiological functions of the FOS family in MM cells, we determined their expression profiles using a panel of MM cell lines and primary MM cells. Among the family members, c‐FOS and its heterodimeric partner in the canonical AP‐1 complex, c‐JUN, were expressed in most cell lines at various levels, whereas FOSB was detected in only a few cell lines (Figure 4B). These results were reproduced in independent analyses using primary samples showing that MM cells expressed *FOS* and *JUN* but not *FOSB* transcripts at higher levels than normal plasma cells and CD138‐positive cells from patients with monoclonal gammopathy of undetermined significance (Figure 4C). Furthermore, the overall survival of MM patients with higher expression of *FOS* was significantly shorter than that of MM patients with lower expression of *FOS* when they were treated with Total Therapy 2/3 (Figure 4). Next, we performed a shRNA‐mediated knockdown of c‐FOS to verify its role in the expression of IKZF1‐target genes in MM cells. As anticipated, shRNA against *FOS* significantly reduced the expression of IKZF1‐targets with co‐binding of IKZF1 and c‐FOS in enhancer/promoter regions, such as *IRF4* and *SLAMF7*, but not the expression of IKZF1‐targets lacking c‐FOS co‐occupancy, such as *BSG* and *CD48* (Figure 4D, left panel). The expression levels of *FOS* were positively and negatively correlated with the expression levels of *IRF4* and *BSG*, respectively, in sh‐FOS‐treated cells (Figure 4D, right panel). These results imply that c‐FOS positively regulates the transcription of a subset of IKZF1‐target genes in MM cells.

### 3.3 c‐FOS is an integral component of the IKZF1 complex in MM cells

**FIGURE 5:**
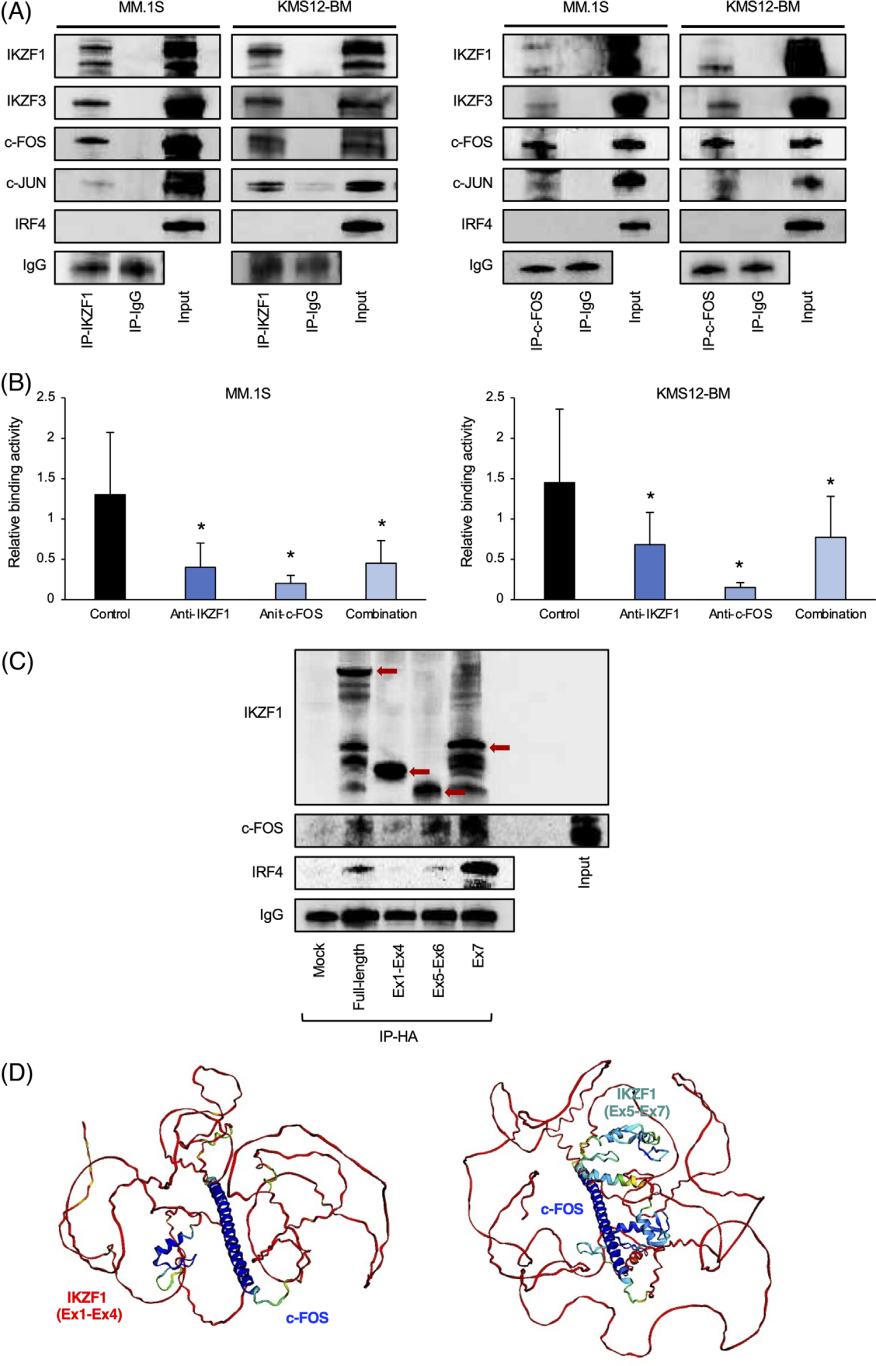
Direct interaction of IKZF1 and c‐FOS in multiple myeloma (MM) cells. (A) Left panel: Nuclear extracts from MM.1S and KMS12‐BM cells were immunoprecipitated with rabbit anti‐IKZF1 antibody or isotype‐matched immunoglobulin (IgG). The immunoprecipitates were analyzed by immunoblotting with specific antibodies against IKZF1, IKZF3, c‐FOS, c‐JUN, IRF4 and rabbit IgG. Input: Immunoblotting of nuclear extracts fractioned before immunoprecipitation. Right panel: The same experiments were carried out with c‐FOS immunoprecipitates. (B) The binding of the IKZF1 complex to oligonucleotides containing an IKZF consensus motif was measured by sandwich immunoassay and is shown as the relative activity against the data obtained in the absence of blocking antibodies. Antibody perturbation was carried out with isotype‐matched immunoglobulin (Control), an anti‐IKZF1 antibody, an anti‐c‐FOS antibody and a combination of the two antibodies. *p* < .05 by Student's *t*‐test against Control (*n* = 5). (C) HEK293T cells were transfected with an empty vector (Mock) or expression vectors carrying HA‐tagged full‐length IKZF1 protein, the exon 1‐exon 4 fragment, the exon 5‐exon 6 fragment, or the exon 7 fragment of IKZF1 together with a FLAG‐tagged c‐FOS expression vector. Nuclear extracts were isolated 24 h after transfection and immunoprecipitated with an anti‐HA antibody, followed by immunoblotting with antibodies against HA tag (IKZF1), FLAG tag (c‐FOS) or rabbit immunoglobulin (IgG). Red arrows denote the positions of the transfected IKZF1 fragments. (D) Structure‐based prediction of IKZF1‐c‐FOS interactions using the AlphaFold2 program.

Co‐occupancy of IKZF1 and c‐FOS on the same consensus in ∼50% of target genes strongly suggests that the two factors form a complex on the myeloma genome. To determine whether IKZF1 and c‐FOS interact physically or bind independently on the same regions, we performed immunoprecipitation‐immunoblot assays using nuclear extracts from two MM cell lines, MM.1S and KMS12‐BM. Immunoprecipitates with an IKZF1‐specific antibody contained c‐FOS and c‐JUN in addition to IKZF3 but not IRF4 (Figure 5A, left panel). Conversely, c‐FOS immunoprecipitates contained IKZF1 and IKZF3 in addition to c‐JUN but not IRF4 (Figure 5A, right panel). Furthermore, an anti‐c‐FOS antibody perturbed the binding of the IKZF complex to the peptide containing a cognate IKZF‐binding motif in a protein‐DNA binding assay, suggesting that IKZF1/3 and c‐FOS form a stable complex on the genome (Figure 5B). Previous studies have shown that the physical interactions of IKZF1 isoforms are essential for the normal development of lymphoid cells.^33^, ^34^ The specification and homeostasis of the lymphatic system are profoundly affected by IKZF1 mutations that impair protein‐protein interactions or alter the ratio of DNA‐binding and non‐DNA‐binding isoforms.^15^, ^34^ To test which domains of the IKZF1 protein are involved in c‐FOS binding, we generated HA‐tagged fragments of different IKZF1 domains and expressed them in HEK293T cells together with FLAG‐tagged c‐FOS. c‐FOS was found to be associated with the activation and dimerization domains, exons 5–6 and exon 7, respectively, of IKZF1 in immunoprecipitation‐immunoblot assays using nuclear extracts from transfected cells (Figure 5C). Consistent with this result, a structure‐based prediction of protein‐protein interactions using the AlphaFold2 program showed the binding of c‐FOS peptides to the region sandwiched between exons 5–6 and exon 7 of IKZF1 (Figure 5D). Forced expression of IKZF1 fragments with the ability of c‐FOS binding induced the expression of IRF4, which is not usually transcribed in HEK293T cells (Figure 5C). Collectively, our data suggest that c‐FOS is an integral component of the IKZF1 complex in MM cells and primarily mediates activator functions of the complex via direct binding to IKZF1 on the myeloma genome.

### 3.4 c‐FOS overexpression is associated with lenalidomide resistance in MM cells

**FIGURE 6:**
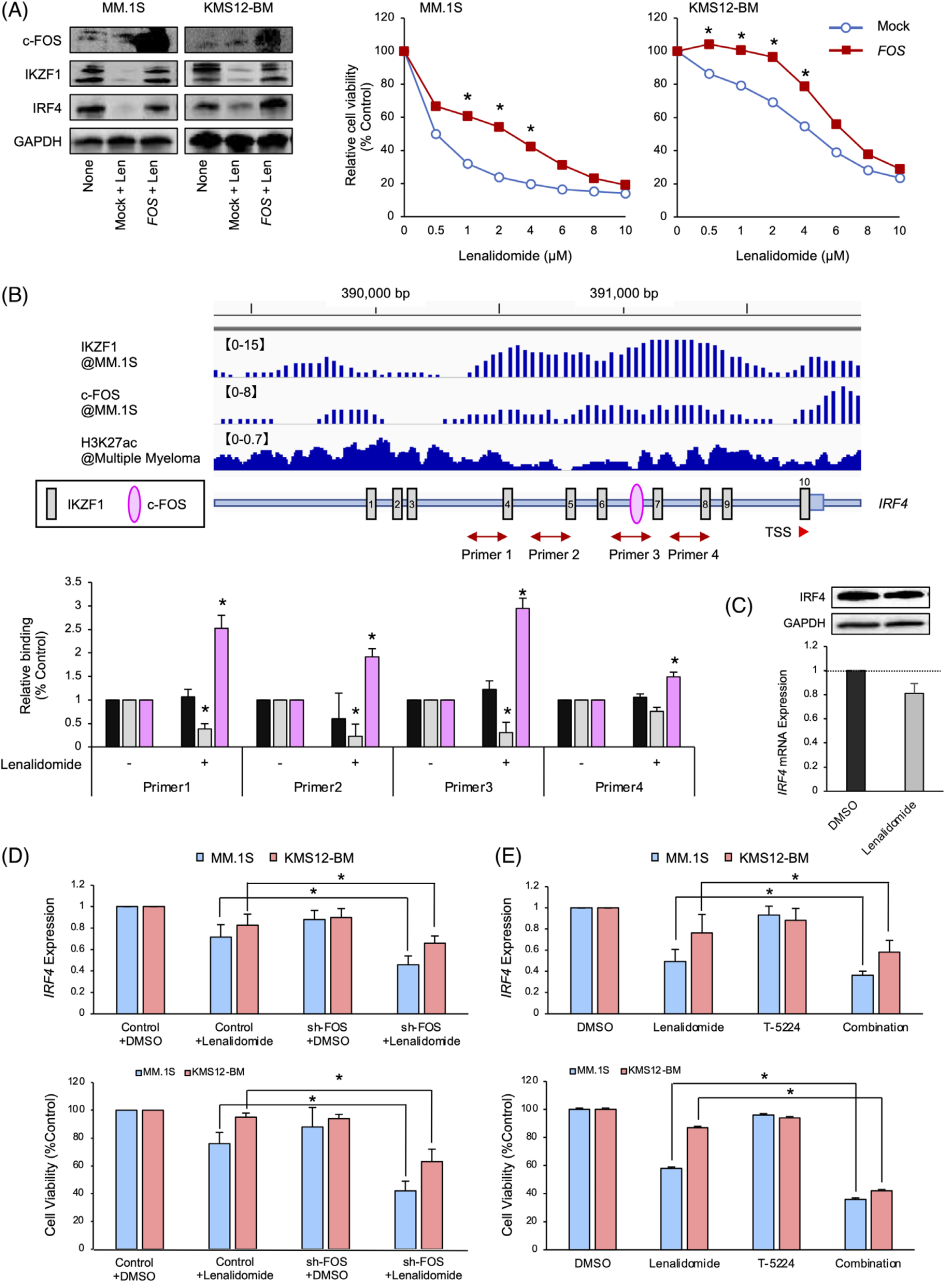
c‐FOS mediates lenalidomide resistance in multiple myeloma (MM) cells. (A) Left panel: MM.1S and KMS12‐BM cells were transfected with c‐FOS expression vector or empty vector (Mock) and treated with the vehicle alone (None) or 2.5 μM lenalidomide for 24 h, followed by immunoblot analysis for the expression of the indicated molecules. Right panel: MM.1S and KMS12‐BM cells were transfected with a c‐FOS expression vector or an empty vector (Mock) and treated with various concentrations of lenalidomide for 72 hours. Cell viability was determined by MTT reduction assay with a Cell Counting Kit (Fujifilm Wako Biochemicals). The graphs show the means of triplicate samples; the S.D. was less than 10% and thus omitted. *p < .05 by one‐way ANOVA with Student–Newman–Keuls multiple comparison tests. (B) Upper panel: Schematic representation of the *IRF4* promoter region from the chromatin immunoprecipitation (ChIP)‐Atlas data. The relative positions of the putative binding sites of transcription factors are approximated by the symbols shown in the box. TSS: transcription start site. Bidirectional red arrows indicate regions that were PCR amplified in ChIP assays. Lower panel: Chromatin suspensions were prepared from KMS12‐BM cells cultured with vehicle alone (DMSO) or 2.5 μM lenalidomide for 24 h and immunoprecipitated with anti‐IKZF1 (grey bars) and c‐FOS (pink bars) antibodies or IgG (back bars). The resulting precipitates were subjected to PCR to amplify the regions shown in the upper panel. *p < .05 against IgG by one‐way ANOVA with Student–Newman–Keuls multiple comparison tests. (C) The expression of IRF4 protein and mRNA in DMSO‐ or lenalidomide‐treated KMS12‐BM cells. (D) MM.1S and KMS12‐BM cells were transfected with sh‐FOS expression vector or empty vector (Control) and treated with vehicle alone (DMSO) or 10 μM lenalidomide. (E) MM.1S and KMS12‐BM cells were treated with vehicle alone (DMSO), 10 μM lenalidomide, 20 μM T‐5224, or the combination of lenalidomide and T‐5224. Upper panels: The expressions of *IRF4* and *GAPDH* transcripts were examined by quantitative real‐time reverse transcription‐PCR after 24 h. The results were normalized to the values of DMSO‐treated cells. Lower panels: Cell viability was determined by MTT reduction assay after 72 h and is shown as the percentage of untreated cells (%Control). The data are presented as the means of three biological replicates with S.D. (bars). *p < .05 by one‐way ANOVA with Student–Newman–Keuls multiple comparison tests.

Lenalidomide exerts anti‐MM activity by inducing the degradation of IKZF1 and the subsequent repression of *IRF4* transcription.^6^, ^7^ Given that c‐FOS mediates the activator function of the IKZF1 complex in MM cells, it is possible that the expression levels of c‐FOS modify the lenalidomide sensitivity of MM cells. To substantiate this hypothesis, we overexpressed c‐FOS in MM.1S and KMS12‐BM cells and found that forced expression of c‐FOS significantly mitigated the cytotoxicity of lenalidomide at clinically relevant concentrations (< 4 μM) along with sustained expression of IRF4 (Figure 6A). c‐FOS overexpression did not considerably affect the baseline expression of IKZF1 in MM cells (Figure S5) despite the existence of c‐FOS‐binding sites in enhancer regions of the *IKZF1* gene.^35^ The transcription factor‐binding motif algorithm TRANSFAC detected 10 IKZF1‐binding sites and a c‐FOS binding site in the *IRF4* promoter. Sites 4−9 were actually occupied by IKZF1 and c‐FOS, and were transcriptionally active, as evidenced by histone H3K27 hyperacetylation in the ChIP‐Atlas analyses (Figure 6B, upper panel). Lenalidomide significantly decreased the amounts of IKZF1 on these sites to ∼20% in KMS12‐BM cells; however, c‐FOS binding to the corresponding sequences was increased in ChIP‐quantitative PCR analyses (Figure 6B, lower panel). An increase in c‐FOS binding to IKZF‐binding sites was also observed in the *SLAMF7* promoter of lenalidomide‐treated KMS12‐BM cells (Figure S6). Because the viability of KMS12‐BM cells was reduced to only 70% with 2.5 μM lenalidomide (Figure 6A, right panel), the discrepancy between the cytotoxic effects and IKZF1 down‐regulation might be due to increased binding of c‐FOS to the IKZF1 consensus and subsequent enhancement of the activity of the residual IKZF1 complex to maintain *IRF4* transcription. In support of this hypothesis, IRF4 expression was decreased only to ∼80% at mRNA and protein levels by lenalidomide treatment in these experiments (Figure 6C). To corroborate the role of c‐FOS in lenalidomide resistance in a physiological context, we established lenalidomide‐resistant sublines of NCI‐H929 cells via continuous exposure of parental cells to the drug. As expected, c‐FOS expression was readily up‐regulated in lenalidomide‐resistant sublines compared with the parental cell line along with the increased expression of IRF4 (Figure S7A). Overall, c‐FOS might be implicated in lenalidomide resistance and could be a therapeutic target for overcoming the resistance to and/or enhancing the anti‐MM action of lenalidomide.

### 3.5 Genetic and pharmacological inhibition of c‐FOS enhances the anti‐myeloma activity of lenalidomide in vitro

To obtain proof of the above concept, we investigated whether disruption of the physical and/or functional interactions of IKZF1 with c‐FOS increases the sensitivity of MM cells to lenalidomide. First, we transduced MM cell lines with shRNA against *FOS* and confirmed a > 60% reduction in the expression of *FOS* mRNA and c‐FOS protein in lenalidomide‐treated MM cells (Figure S8A,B). The shRNA‐mediated c‐FOS down‐regulation significantly exacerbated the lenalidomide‐induced decrease in the expression levels of *IRF4* transcripts as well as diminished cell viability in MM.1S and KMS12‐BM cells (Figure 6D and Figure S8C). Next, we carried out the same experiments using the selective AP‐1 inhibitor T‐5224 (3‐{5‐[4‐(cyclopentyloxy)−2‐hydroxybenzoyl]−2‐[(3‐hydroxy‐1,2‐benzisoxazol‐6‐yl)methoxy]phenyl} propionic acid). This compound was reported to inhibit AP‐1 activity via interference with the physical interaction of c‐FOS and DNA without altering the abundance of c‐FOS/c‐JUN^36^, ^37^, ^38^; however, T‐5224 modestly decreased the expression levels of c‐FOS in our experiments (Figure S7B). Treatment of MM cells with T‐5224 alone did not affect the expression of *IRF4* or cell viability but significantly enhanced the cytotoxicity of lenalidomide in both cell lines (Figure 6E). Furthermore, lenalidomide and T‐5224 showed additive/synergistic effects in isobologram analysis of drug combinations in MM.1S cells and at least additive effects in KMS12‐BM cells (Figure S9). The combined effects were less prominent in KMS12‐BM cells treated with higher doses of lenalidomide because of the concave pattern of the drug response (Figure 6A, right panel). In addition, we confirmed the combined cytotoxic effects of lenalidomide and T‐5224 in patient‐derived MM cells (Figure S10). We also evaluated whether lenalidomide resistance is attenuated by the combination with T‐5224 in NCI‐H929LR cells. As expected, lenalidomide alone did not reduce the expression level of IRF4 or cell viability at all in lenalidomide‐resistant NCI‐H929 sublines, but the addition of T‐5224 apparently overcame lenalidomide resistance as indicated by decreased IRF4 expression and cell viability (Figure S7B,C).

### 3.6 A selective AP‐1 inhibitor enhances the anti‐myeloma activity of lenalidomide in vivo

**FIGURE 7:**
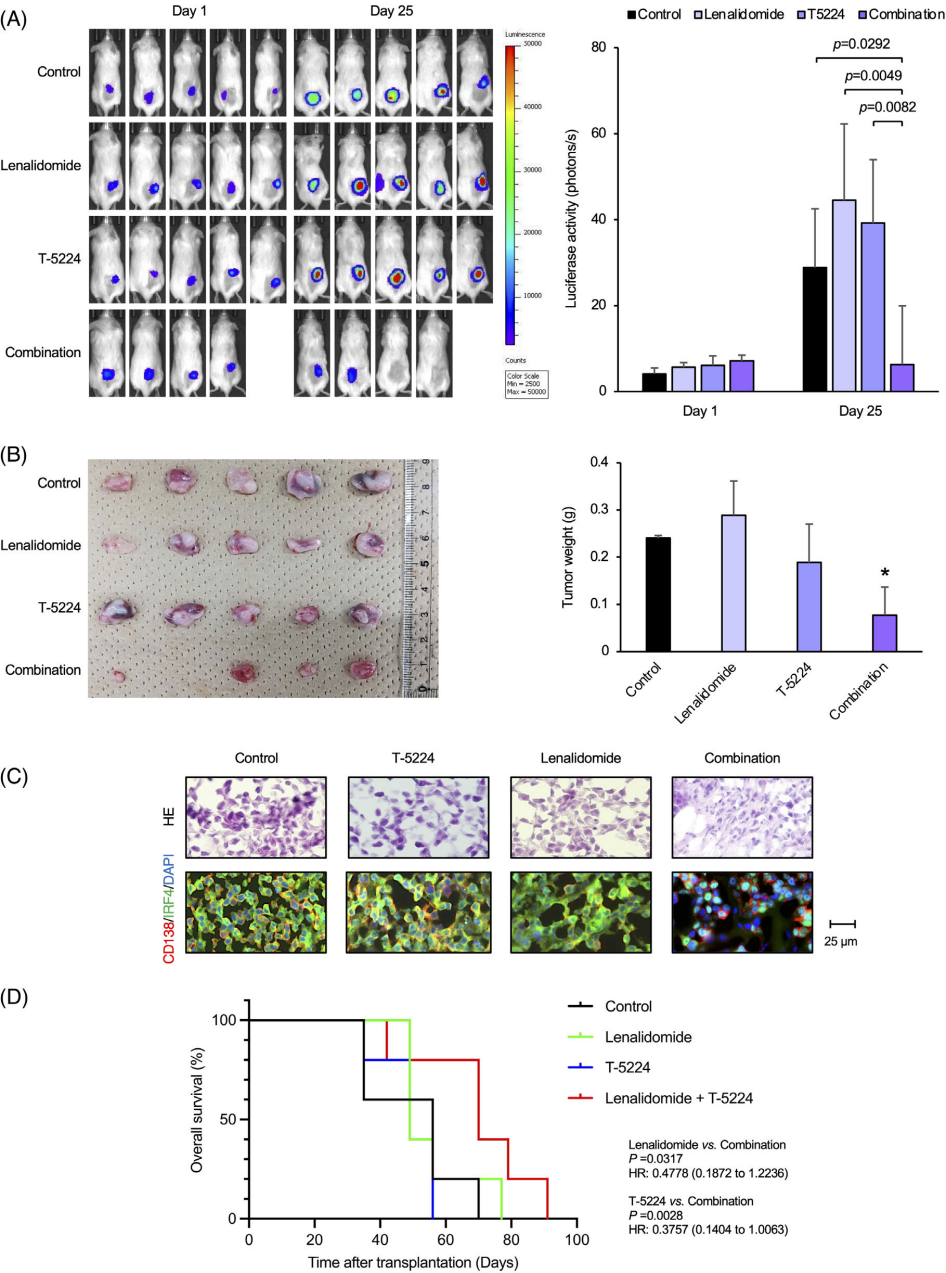
A selective activator protein‐1 (AP‐1) inhibitor augments the effects of lenalidomide in murine multiple myeloma (MM) models. (A) Luciferase‐expressing MM.1S cells were inoculated subcutaneously into the right thigh of NOD/SCID mice (1 × 10^6^ cells/mouse). When the inoculated tumour became measurable (defined as day 1), we started treatment with vehicle alone (0.9% NaCl, twice a week), 20 mg/kg T‐5224 (twice a week), 10 mg/kg lenalidomide (twice a week), or the combination of T‐5224 and lenalidomide (20 mg/kg T‐5224 and 10 mg/kg lenalidomide, twice a week) intraperitoneally for two weeks. Left panel: Representative photographs of mice on day 1 and day 25 (original magnification: 2×). Right panel: Quantitative data of *ex vivo* bioluminescence imaging on day 1 and day 25 expressed in photons/s (photons/s). The mean ± S.D. (bars) is shown. *p‐*Values were determined by one‐way ANOVA with Student–Newman–Keuls multiple comparison test. (B) Left panel: Representative photographs of tumours resected on day 25 (original magnification: ×2). Right panel: The mean ± S.D. (bars) of tumour weight. *p < .05 by one‐way ANOVA with Student–Newman–Keuls multiple comparison tests. (C) Upper panel: Tumor sections were prepared from mice and stained with hematoxylin and eosin (HE). Lower panel: Serial tumour sections were immunostained for IRF4 (green) and CD138 (red), followed by counterstaining with DAPI (blue). Merged images are shown at the bottom. The data are representative of several independent experiments. (D) We transplanted luciferase‐expressing KMS26 cells (1 × 10^6^ cells/mouse) into BALB/c mice via tail‐vein injection. After confirming the engraftment of transplanted cells on day 14 via ex vivo luciferase assays, we assigned recipients to four groups (*n* = 5), which were standardized on the basis of total luciferase activity, and started the treatments with vehicle alone (0.9% NaCl, three times a week), 20 mg/kg T‐5224 (three times a week), 10 mg/kg lenalidomide (three times a week), or the combination of T‐5224 and lenalidomide (20 mg/kg T‐5224 and 10 mg/kg lenalidomide, three times a week) intraperitoneally for 3 weeks. *p*‐Values and hazard ratios (95% confidential interval) were calculated by log‐rank test between the combined treatment group (red) and the lenalidomide‐alone (green) or T‐5224‐alone treatment group (blue). No significant differences were observed between the vehicle‐treated group (Control) and the lenalidomide‐alone or T‐5224 alone‐treatment group.

Finally, we attempted to replicate the combined effects of lenalidomide and T‐5224 in a murine MM model, in which luciferase‐expressing sublines of MM.1S were subcutaneously inoculated into NOD/SCID mice.^25^, ^26^, ^27^ When measurable tumours developed, mice were randomly assigned to four groups (*n* = 5 per group) and treated with vehicle alone (0.9% NaCl), T‐5224 alone, lenalidomide alone, or lenalidomide plus T‐5224. The combination of lenalidomide and T‐5224 significantly slowed the growth of inoculated tumours, as evidenced by the decrease in *ex vivo* luciferase activity (Figure 7A and Figure S11) and in the sizes of the tumours resected on day 25 (Figure 7B), whereas T‐5224 or lenalidomide alone did not affect the growth of MM cells at the dose and schedule used in this experiment. Immunofluorescent staining revealed a marked decrease in cell numbers and IRF4 expression in inoculated tumours from the combined treatment group but not in those from other groups (Figure 7C). We also investigated the efficacy of the two drugs in an orthotopic MM model, in which luciferase‐expressing KMS26 cells were transplanted into NOD/SCID mice via tail‐vein injection and engrafted in bone marrow after 14 days on average. After confirming engraftment by measuring *ex vivo* luciferase activity, we divided recipients into four groups (*n* = 5), in which the sum of luciferase activity was not statistically different (vehicle‐treated group; 3.58 ± 1.34, lenalidomide‐treated group; 4.04 ± 0.37, T‐5224‐treated group; 5.09 ± 0.78, lenalidomide and T‐5224‐treated group; 4.03 ± 0.47 × 10^4^ photons/s, *P* = 0.2327 by one‐way ANOVA), and started the treatments with vehicle alone, T‐5224 alone, lenalidomide alone, or the combination of lenalidomide and T‐5224. As shown in Figure 7D, treatment with lenalidomide or T‐5224 failed to prolong the survival of KMS26‐transplanted mice; however, the combination of the two agents led to a significant increase in overall survival compared with the single‐agent therapies. No obvious toxicities were observed in any group of mice (data not shown).

## 4 DISCUSSION

**FIGURE 8:**
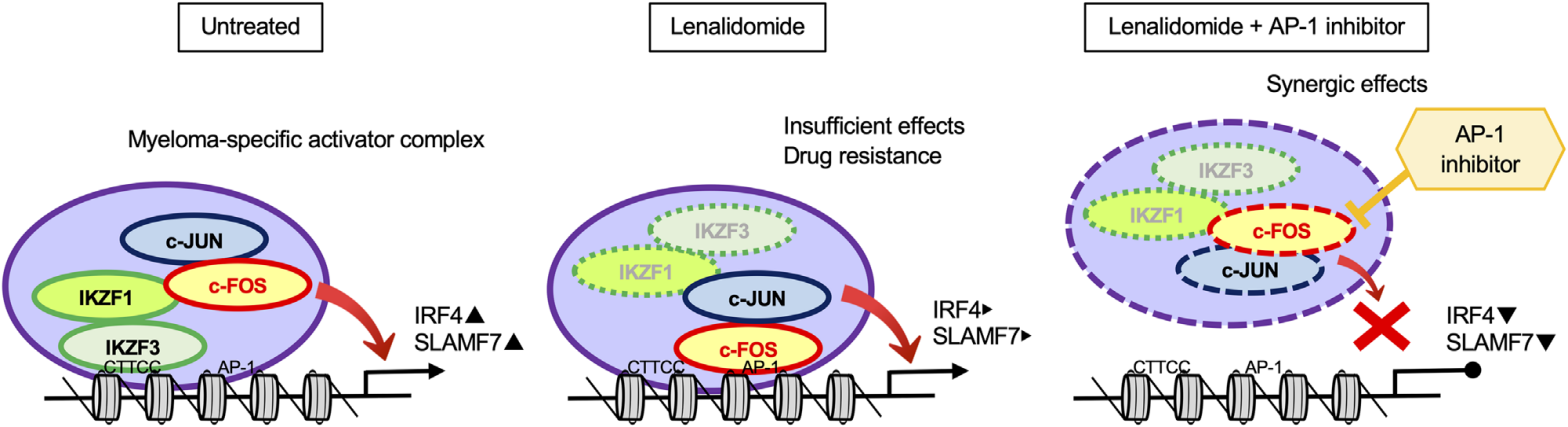
Graphical abstract. c‐FOS, a subunit of the activator protein‐1 (AP‐1) transactivator, is an integral component of the IKZF1 complex and is primarily responsible for the activator function of the complex in multiple myeloma (MM) cells. Left panel: The IKZF complex binds to the enhancer/promoter regions of the genes involved in the growth and survival of MM cells such as *IRF4* and *SLAMF7* through the canonical IKZF‐binding motif CTTCC with c‐FOS/c‐JUN. Middle panel: Lenalidomide induces ubiquitin‐dependent degradation of IKZF1/IKZF3; however, residual c‐FOS, the level of which is often increased by lenalidomide treatment, binds to the AP‐1 consensus sequences, which present in the vicinity of IKZF‐binding sites of certain genes including *IRF4* and *SLAMF7*, leading to sustained expression of these genes and lenalidomide resistance. Right panel: A selective AP‐1 inhibitor, T‐5224, binds to the DNA‐binding domain of c‐FOS and mitigates the residual activity of the MM‐specific activator complex, resulting in complete *IRF4* down‐regulation and augmentation of the anti‐MM effects of lenalidomide.

Lenalidomide exerts pharmacological action through the ubiquitin‐dependent degradation of IKZF1 and subsequent down‐regulation of IRF4, a critical factor for the survival of MM cells.^6^, ^7^, ^8^ IKZF1 functions as a tumour suppressor via transcriptional repression of oncogenes in normal hematopoietic lineages,^13^, ^14^, ^15^ whereas it activates *IRF4* and other oncogenes in MM cells.^16^, ^17^, ^18^ These results suggest that the IKZF1 complex switches its function from transcriptional repressor to activator in MM cells; however, little is known about the underlying mechanisms of this switch. In this study, using bioinformatic and biological approaches, we determined that c‐FOS, a bona fide transcriptional activator of the AP‐1 family, is an integral component of the IKZF1 complex and is primarily responsible for the activator function of the complex in MM cells. Because the binding sequence of c‐FOS on the myeloma genome is different from common AP‐1‐binding motifs, such as TPA‐responsive elements and cAMP‐responsive elements,^19^, ^20^ it is highly possible that c‐FOS binds to DNA through protein‐protein interactions with IKZF1. This is the first report of the complex formation of IKZF1 and c‐FOS as well as the disease‐specific functions of this complex (Figure 8, left panel). The MM‐specific IKZF complex is composed of IKZF1/IKZF3 and c‐FOS/c‐JUN but does not contain IRF4, in contrast to a previous finding that IRF4 is a component of the IKZF complex in normal plasma cells.^39^ The IKZF‐IRF4 complex is associated with the NuRD complex to mediate repressor functions during normal plasma cell differentiation.^39^ The exclusion of IRF4 might be involved in the switch of the IKZF complex from a repressor to a transcriptional activator during plasma cell transformation.

AP‐1 is a transcription factor complex composed of subunit proteins from the FOS (c‐FOS, FOSB, FRA‐1 and FRA‐2), JUN (c‐JUN, JUNB and JUND), ATF and MAF subfamilies, with a c‐FOS/c‐JUN heterodimer as a representative complex.^19^, ^20^ Each subunit carries basic leucine zipper domains, which promote dimerization via hydrophobic stretches and interactions with DNA via positively charged amino acids.^19^ AP‐1 regulates the proliferation, differentiation, and cell death of various cell types, including MM cells.^20^ For example, it has been reported that c‐FOS/c‐JUN activity is altered in almost all MM patients and plays a major role in a transcriptional network associated with myeloma survival.^40^ Fan et al. demonstrated that JUNB facilitates the proliferation, survival, and drug resistance of MM cells in the bone marrow microenvironment.^41^, ^42^ Furthermore, Ohguchi et al. identified c‐FOS as a crucial factor for MM cell growth and survival downstream of the histone demethylase 6B‐MAP kinase pathways.^43^ AP‐1‐binding motifs are present in enhancers of several genes whose activity is increased in MM cells, and c‐FOS binding was actually detected at the *MAF* gene promoter.^44^, ^45^ Consistent with the unique role of AP‐1 in MM biology, Liu et al. identified AP‐1‐overexpressing subpopulations in MM patients using single‐cell genomics.^46^ Although the drug sensitivity of AP‐1^high^ subclones was not described in their study, this subpopulation seems to possess malignant characteristics because they appear during disease progression or relapse.^46^

Given the principal role of c‐FOS in the activator function of the IKZF1 complex in MM cells, the sole targeting of IKZF1/IKZF3 by lenalidomide might be insufficient for inducing the complete down‐regulation of *IRF4* expression and subsequent cell death because residual AP‐1 activity could sustain the transcription of *IRF4* (Figure 8, middle panel). Similarly, an increase in c‐FOS expression was observed in lenalidomide‐treated non‐small‐cell lung cancer cells and is speculated to be a mechanism underlying the relative insensitivity of lung cancer to lenalidomide.^47^ Considering these findings, the addition of AP‐1 inhibitors may be a strategy for overcoming lenalidomide resistance and/or augmenting the efficacy of lenalidomide. In this research, we demonstrated that a selective AP‐1 inhibitor, T‐5224, significantly enhanced the anti‐MM activity of lenalidomide in vitro and in two murine MM models (Figure 8, right panel). Among AP‐1 inhibitors, T‐5224 is a lead compound that specifically inhibits the binding of AP‐1 to regulatory regions of target genes, such as inflammation‐related genes,^36^, ^37^ and is currently under phase II clinical trials for patients with rheumatoid arthritis and other inflammatory diseases after the absence of serious side effects was confirmed in phase I trials (JapicCTI‐101359).^38^ The clinical relevance of AP‐1 inhibitors is supported by a recent report that T‐5224 was able to restore the sensitivity of acute myeloid leukaemia cells to cytosine arabinoside.^48^ The spectrum of clinical applications of AP‐1 inhibitors might be expanded to MM in combination with lenalidomide in the future.

Finally, it should be noted that c‐FOS may be involved in high‐risk features of MM. For example, c‐FOS is known to be positively regulated by the histone H3K36 methyltransferase MMSET/NSD2, which is ectopically expressed due to the chromosome translocation t(4;14) and might contribute to the overexpression of *IRF4* and *SLAMF7* as a direct transactivator in high‐risk MM with t(4;14).^49^, ^50^ Lenalidomide resistance is a well‐characterized clinical feature of MM patients with t(4;14)^51^, ^52^ and might be mediated via c‐FOS overexpression under the influence of MMSET. According to Annunziata et al., the shRNA‐mediated knockdown of MMSET reduces c‐FOS expression in t(4;14)‐harbouring MM cells.^45^ These results suggest that c‐FOS may be an important therapeutic target in high‐risk MM patients, who are usually highly resistant to lenalidomide.^4^, ^5^

## 5 CONCLUSIONS

In conclusion, c‐FOS determines lenalidomide sensitivity and mediates drug resistance in MM cells as a co‐factor of IKZF1 and thus, could be a novel therapeutic target for further improvement of the prognosis of MM patients.

## CONFLICT OF INTEREST STATEMENT

The authors declare no conflict of interest.

## Supporting information

Supporting Information

## Data Availability

The ChIP‐seq data were deposited in the MINSEQE‐compliant GEO database under accession numbers GSE194381 and GSE194387.

## References

[1] Furukawa Y , Kikuchi J . Molecular basis of clonal evolution in multiple myeloma. Int J Hematol. 2020;111:496‐511. 32026210 10.1007/s12185-020-02829-6

[2] Suzuki K . Latest treatment strategies aiming for a cure in transplant‐eligible multiple myeloma patients: how I cure younger MM patients with lower cost. Int J Hematol. 2020;111:512‐518. 32125606 10.1007/s12185-020-02841-w

[3] Leleu X , Martin T , Weisel K , et al. Anti‐CD38 antibody therapy for patients with relapsed/refractory multiple myeloma: differential mechanisms of action and recent clinical trial outcomes. Ann Hematol. 2022;101:2123‐2137. 35943588 10.1007/s00277-022-04917-5 PMC9463192

[4] Holstein SA , Suman VJ , McCarthy PL . Update on the role of lenalidomide in patients with multiple myeloma. Ther. Adv Hematol. 2018;9:175‐190. 10.1177/2040620718775629 PMC6041862 30013765

[5] Moreau P , Zamagni E , Mateos M‐V . Treatment of patients with multiple myeloma progressing on frontline‐therapy with lenalidomide. Blood Cancer J. 2019;9:38. 30894516 10.1038/s41408-019-0200-1 PMC6426995

[6] Ito T , Yamaguchi Y , Handa H . Exploring ubiquitin ligase cereblon as a target for small‐molecule compounds in medicine and chemical biology. Cell Chem Biol. 2021;28:987‐999. 34033753 10.1016/j.chembiol.2021.04.012

[7] Bjorklund CC , Lu L , Kang J , et al. Rate of CRL4^CRBN^ substrate Ikaros and Aiolos degradation underlies differential activity of lenalidomide and pomalidomide in multiple myeloma cells by regulation of c‐Myc and IRF4. Blood Cancer J. 2015;5: e354. 26430725 10.1038/bcj.2015.66 PMC4635186

[8] Ikeda S , Kitadate A , Abe F , et al. Hypoxia‐inducible microRNA‐210 regulates the DIMT1‐IRF4 oncogenic axis in multiple myeloma. Cancer Sci. 2017;108:641‐652. 28164410 10.1111/cas.13183 PMC5406542

[9] Gooding S , Ansari‐Pour N , Towfic F , et al. Multiple cereblon genetic changes are associated with acquired resistance to lenalidomide or pomalidomide in multiple myeloma. Blood. 2021;137:232‐237. 33443552 10.1182/blood.2020007081 PMC7893409

[10] Haertle L , Barrio S , Munawar U , et al. Cereblon enhancer methylation and IMiDs resistance in multiple myeloma. Blood. 2021;138:1721‐1726. 34115836 10.1182/blood.2020010452 PMC8569411

[11] Jones JR , Barber A , Le Bihan Y‐V , et al. Mutations in CRBN and other cereblon pathway genes are infrequently associated with acquired resistance to immunomodulatory drugs. Leukemia. 2021;35:3017‐3020. 34373585 10.1038/s41375-021-01373-4 PMC8478640

[12] John LB , Ward AC . The Ikaros gene family: Transcriptional regulators of hematopoiesis and immunity. Mol Immunol. 2011;48:1272‐1278. 21477865 10.1016/j.molimm.2011.03.006

[13] Georgopoulos K . The making of a lymphocyte: the choice among disparate cell fates and the IKAROS enigma. Genes Dev. 2017;31:439‐450. 28385788 10.1101/gad.297002.117 PMC5393059

[14] Ding Y , Zhang B , Payne J , et al. Ikaros tumour suppressor function includes induction of active enhancers and super‐enhancers along with pioneering activity. Leukemia. 2019;33:2720‐2731. 31073152 10.1038/s41375-019-0474-0 PMC6842075

[15] Heizmann B , Kastner P , Chan S . The Ikaros family in lymphocyte development. Curr Opin Immunol. 2018;51:14‐23. 29278858 10.1016/j.coi.2017.11.005

[16] Lopez‐Girona A , Heintel D , Zhang LH , et al. Lenalidomide downregulates the cell survival factor, interferon regulatory factor‐4, providing a potential mechanistic link for predicting response. Br J Haematol. 2011;154:325‐336. 21707574 10.1111/j.1365-2141.2011.08689.x

[17] Zhu YX , Braggio E , Shi C‐X , et al. Identification of cereblon‐binding proteins and relationship with response and survival after IMiDs in multiple myeloma. Blood. 2014;124:536‐545. 24914135 10.1182/blood-2014-02-557819 PMC4110660

[18] Fedele PL , Willis SN , Liao Y , et al. IMiDs prime myeloma cells for daratumumab‐mediated cytotoxicity through loss of Ikaros and Aiolos. Blood. 2018;132:2166‐2178. 30228232 10.1182/blood-2018-05-850727

[19] Bejjani F , Evanno E , Zibara K , Piechaczyk M . Jariel‐Encontre I. The AP‐1 transcriptional complex: Local switch or remote command? Biochim Biophys Acta Rev Cancer. 2019;1872:11‐28. 31034924 10.1016/j.bbcan.2019.04.003

[20] Fan F , Podar K . The role of AP‐1 transcription factor in plasma cell biology and multiple myeloma pathophysiology. Cancers. 2021;13:2326. 34066181 10.3390/cancers13102326 PMC8151277

[21] Koyama D , Kikuchi J , Kuroda Y , Ohta M , Furukawa Y . AMP‐activated protein kinase activation primes cytoplasmic translocation and autophagic degradation of the BCR‐ABL protein in CML cells. Cancer Sci. 2021;112:194‐204. 33070465 10.1111/cas.14698 PMC7780059

[22] Saito S , Kikuchi J , Koyama D , et al. Eradication of central nervous system leukemia of T‐cell origin with a brain‐permeable LSD1 inhibitor. Clin Cancer Res. 2019;25:1601‐1611. 30518632 10.1158/1078-0432.CCR-18-0919 PMC6397674

[23] Zhou X , Medina S , Bolt AM , et al. Inhibition of red blood cell development by arsenic‐induced disruption of GATA‐1. Sci Rep. 2020;10: 19055. 33149232 10.1038/s41598-020-76118-x PMC7643154

[24] Molnár Á , Georgopoulos K . The Ikaros gene encodes a family of functionally diverse zinc finger DNA‐binding proteins. Mol Cell Biol. 1994;14:8292‐8303. 7969165 10.1128/mcb.14.12.8292-8303.1994 PMC359368

[25] Kikuchi J , Koyama D , Wada T , et al. Phosphorylation‐mediated EZH2 inactivation promotes drug resistance in multiple myeloma. J Clin Invest. 2015;125:4375‐4390. 26517694 10.1172/JCI80325 PMC4665777

[26] Kikuchi J , Kuroda Y , Koyama D , et al. Myeloma cells are activated in bone marrow microenvironment by the CD180/MD‐1 complex which senses lipopolysaccharide. Cancer Res. 2018;78:1766‐1778. 29363546 10.1158/0008-5472.CAN-17-2446

[27] Kikuchi J , Kodama N , Takeshita M , et al. EMD originates from hyaluronan‐induced homophilic interactions of CD44 variant‐expressing MM cells under shear stress. Blood Adv. 2023;7:508‐524. 35930695 10.1182/bloodadvances.2022007291 PMC9979770

[28] Kikuchi J , Hori M , Sorimachi N , et al. Soluble SLAMF7 promotes the growth of myeloma cells via homophilic interaction with surface SLAMF7. Leukemia. 2020;34:180‐195. 31358854 10.1038/s41375-019-0525-6

[29] Eichner R , Heider M , Fernández‐Sáiz V , et al. Immunomodulatory drugs disrupt the cereblon‐CD147‐MCT1 axis to exert antitumour activity and teratogenicity. Nat Med. 2016;22:735‐743. 27294876 10.1038/nm.4128

[30] Oki S , Ohta T , Shioi G , et al. ChIP‐Atlas: a data‐mining suite powered by full integration of public ChIP‐seq data. EMBO Rep. 2018; 19: e46255. 30413482 10.15252/embr.201846255 PMC6280645

[31] Osada N , Kikuchi J , Koyama D , et al. mTOR inhibitors sensitize multiple myeloma cells to venetoclax via IKZF3‐ and Blimp‐1‐mediated BCL‐2 upregulation. Haematologica. 2021;106:3008‐3013. 34261294 10.3324/haematol.2021.278506 PMC8561283

[32] Kassambara A , Réme T , Jourdan M , et al. GenomicScape: An easy‐to‐use web tool for gene expression data analysis. Application to investigate the molecular events in the differentiation of B cells into plasma cells. PLoS Comput Biol. 2015;11: e1004077. 25633866 10.1371/journal.pcbi.1004077 PMC4310610

[33] Read KA , Jones DM , Freud AG , Oestreich KJ . Established and emergent roles for Ikaros transcription factors in lymphoid cell development and function. Immunol Rev. 2021;300:82‐99. 33331000 10.1111/imr.12936 PMC8015388

[34] Sun L , Liu A , Georgopoulos K . Zinc finger‐mediated protein interactions modulate Ikaros activity, a molecular control of lymphocyte development. EMBO J. 1996;15:5358‐5369. 8895580 PMC452279

[35] Yang L , Luo Y , Wei J . Integrative genomic analyses on Ikaros and its expression related to solid cancer prognosis. Oncol Rep. 2010;24:571‐577. 20596648 10.3892/or_00000894

[36] Aikawa Y , Morimoto K , Yamamoto T , et al. Treatment of arthritis with a selective inhibitor of c‐Fos/activator protein‐1. Nat Biotechnol. 2008; 26:817‐823. 18587386 10.1038/nbt1412

[37] Avouac J , Palumbo K , Tomcik M , et al. Inhibition of activator protein 1 signaling abrogates transforming growth factor β‐mediated activation of fibroblasts and prevents experimental fibrosis. Arthritis Rheum. 2012;64:1642‐1652. 22139817 10.1002/art.33501

[38] Ye N , Ding Y , Wild C , Shen Q , Zhou J . Small molecule inhibitors targeting activator protein 1 (AP‐1). J Med Chem. 2014;57:6930‐6948. 24831826 10.1021/jm5004733 PMC4148154

[39] Ochiai K , Kondo H , Okamura Y , et al. Zinc finger‐IRF composite elements bound by Ikaros/IRF4 complexes function as gene repression in plasma cells. Blood Adv. 2018;2:883‐894. 29669755 10.1182/bloodadvances.2017010413 PMC5915997

[40] Miannay B , Minvielle S , Roux O , et al. Logic programming reveals alteration of key transcription factors in multiple myeloma. Sci Rep. 2017; 7:9257. 28835615 10.1038/s41598-017-09378-9 PMC5569101

[41] Fan F , Bashari MH , Morelli E , et al. The AP‐1 transcription factor JunB is essential for multiple myeloma cell proliferation and drug resistance in the bone marrow microenvironment. Leukemia. 2017;31:1570‐1581. 27890927 10.1038/leu.2016.358

[42] Fan F , Malvestiti S , Vallet S , et al. JunB is a key regulator of multiple myeloma bone marrow angiogenesis. Leukemia. 2021;35:3509‐3525. 34007044 10.1038/s41375-021-01271-9 PMC8632680

[43] Ohguchi H , Harada T , Sagawa M , et al. KDM6B modulates MAPK pathway mediating multiple myeloma cell growth and survival. Leukemia. 2017;31:2661‐2669. 28487543 10.1038/leu.2017.141 PMC5681448

[44] Jin Y , Chen K , De Paepe A , et al. Active enhancer and chromatin accessibility landscape chart the regulatory network of primary multiple myeloma. Blood. 2018;131:2138‐2150. 29519805 10.1182/blood-2017-09-808063 PMC6014038

[45] Annunziata CM , Hernandez L , Davis RE , et al. A mechanistic rationale for MEK inhibitor therapy in myeloma based on blockage of MAF oncogenic expression. Blood. 2011;117:2396‐2404. 21163924 10.1182/blood-2010-04-278788 PMC3062408

[46] Liu R , Gao Q , Foltz SM , et al. Co‐evolution of tumour and immune cells during progression of multiple myeloma. Nat Commun. 2021;12:2559. 33963182 10.1038/s41467-021-22804-x PMC8105337

[47] Kim K , An S , Cha HJ , et al. Lenalidomide induces apoptosis and alters gene expression in non‐small cell lung cancer cells. Oncol Lett. 2013;5:588‐592. 23420263 10.3892/ol.2012.1054 PMC3573063

[48] Wang H , Zhan H , Jiang X , et al. A novel miRNA restores the chemosensitivity of AML cells through targeting FosB. Front Med. 2020;7: 582923. 10.3389/fmed.2020.582923 PMC7573296 33123543

[49] Garlisi CG , Uss AS , Xiao H , et al. A unique mRNA initiated within a middle intron of WHSC1/MMSET encodes a DNA binding protein that suppresses human IL‐5 transcription. Am J Respir Cell Mol Biol. 2001;24:90‐98. 11152655 10.1165/ajrcmb.24.1.4224

[50] Yang P , Guo L , Duan ZJ , et al. Histone methyltransferase NSD2/MMSET mediates constitutive NF‐κB signaling for cancer cell proliferation, survival, and tumour growth via feed‐forward loop. Mol Cell Biol. 2012;32:3121‐3131. 22645312 10.1128/MCB.00204-12 PMC3434505

[51] Yoshida T , Ri M , Fujinami H , et al. Impact of chromosomal abnormalities on the efficacy of lenalidomide plus dexamethasone treatment in patients with relapsed/refractory multiple myeloma. Int J Hematol. 2019;110:228‐236. 31119611 10.1007/s12185-019-02669-z

[52] Pawlyn C , Cairns D , Kaiser M , et al. The relative importance of factors predicting outcome for myeloma patients at different ages: results from 3894 patients in the Myeloma XI trial. Leukemia. 2020;34:604‐612. 31611625 10.1038/s41375-019-0595-5 PMC7214257

[53] Zhan F , Barlogie B , Arzoumanian V , et al. Gene‐expression signature of benign monoclonal gammopathy evident in multiple myeloma is linked to good prognosis. Blood. 2007;109:1692‐1700. 17023574 10.1182/blood-2006-07-037077 PMC1794073

