# Supplementary material for: c‐FOS is an integral component of the IKZF1 transactivator complex and mediates lenalidomide resistance in multiple myeloma
Source: Clin Transl Med. 2023 Aug 15;13(8):e1364. doi: 10.1002/ctm2.1364 (PMC10426395; doi:10.1002/ctm2.1364)
Supplement: Supplementary file 1 — Supporting Information [file CTM2-13-e1364-s001.docx]

**
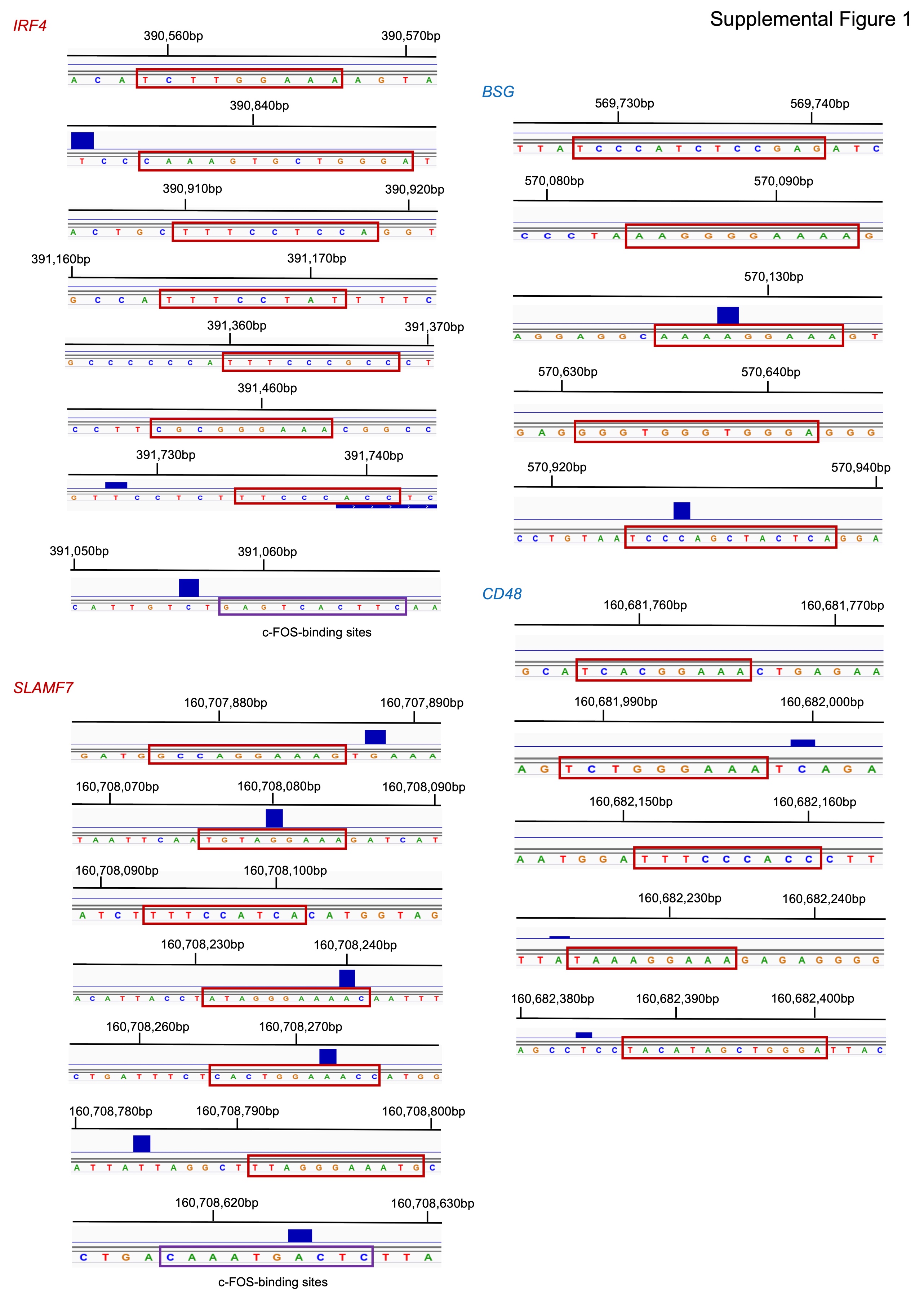
**

**Supplemental Figure 1. ChIP-Atlas search for transcription factors co-bound to IKZF1-binding sites in MM cells.** We incorporated the results of ChIP-seq analyses of IKZF1 binding into the ChIP-Atlas platform and investigated the co-occupancy of IKZF1 with other DNA-binding factors on the genome of MM.1S cells. IKZF1- and c-FOS-binding motifs are enclosed in red and blue boxes, respectively. The blue squares denote the binding of cognate transcription factors to the consensus sequences.

**
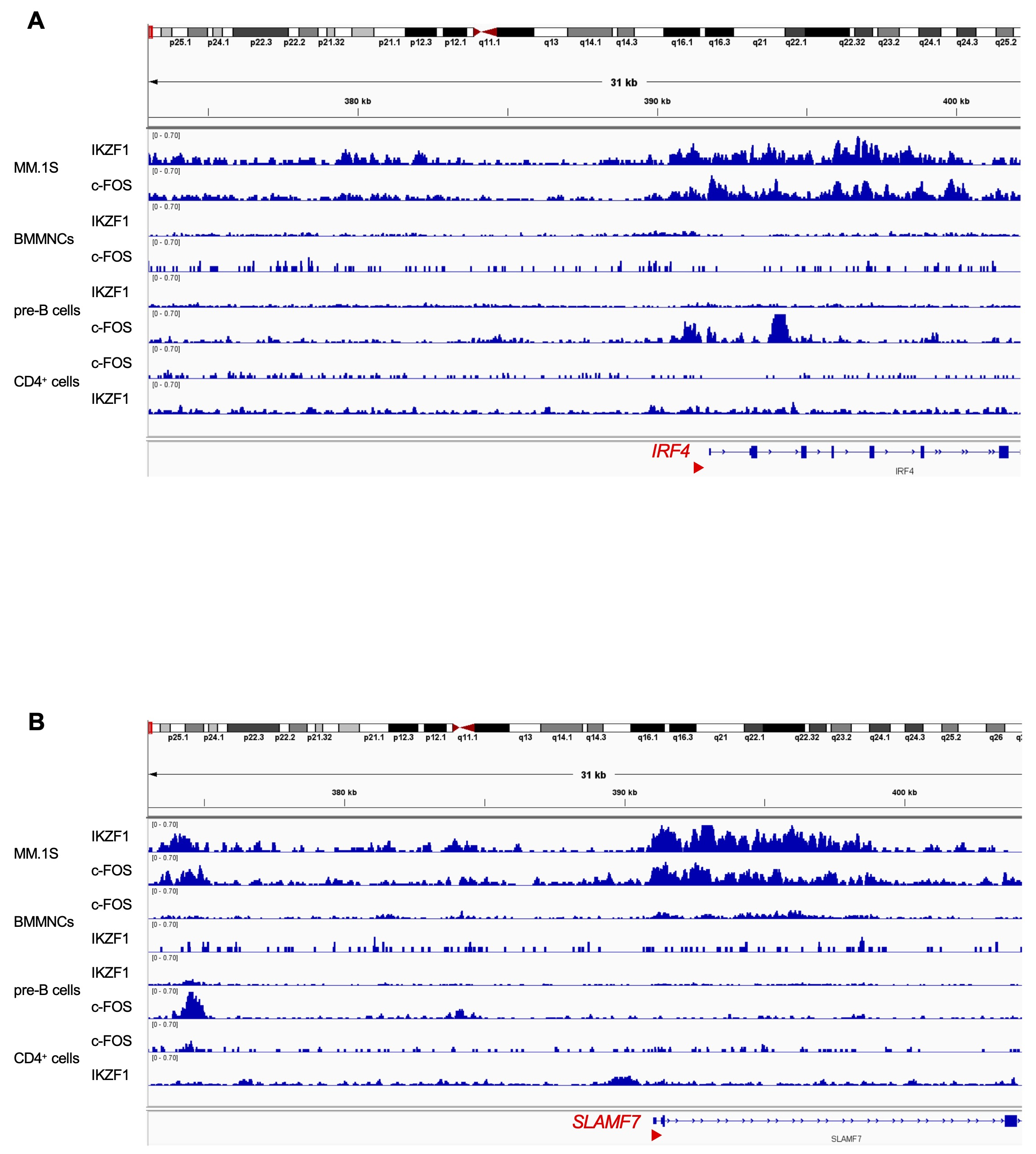
**

**Supplemental Figure 2. The binding of IKZF-1 and c-FOS on the regulatory regions of *IRF4* and *SLAMF7* genes in various hematopoietic cells.** The binding of IKZF1 and c-FOS on promoter/enhancer regions of the *IRF4* **(A)** and *SLAMF7* **(B)** genes are visualized on the ChIP-Atlas platform. We used the data of ChIP-seq of the following cell types: MM.1S, the myeloma cell line MM.1S; BMMNCs, bone marrow mononuclear cells isolated from healthy volunteers; pre-B cells, precursor B-lymphocytes isolated from tonsillectomy specimens; CD4^+^ cells, CD4-positive T-lymphocytes isolated from peripheral blood of healthy volunteers.


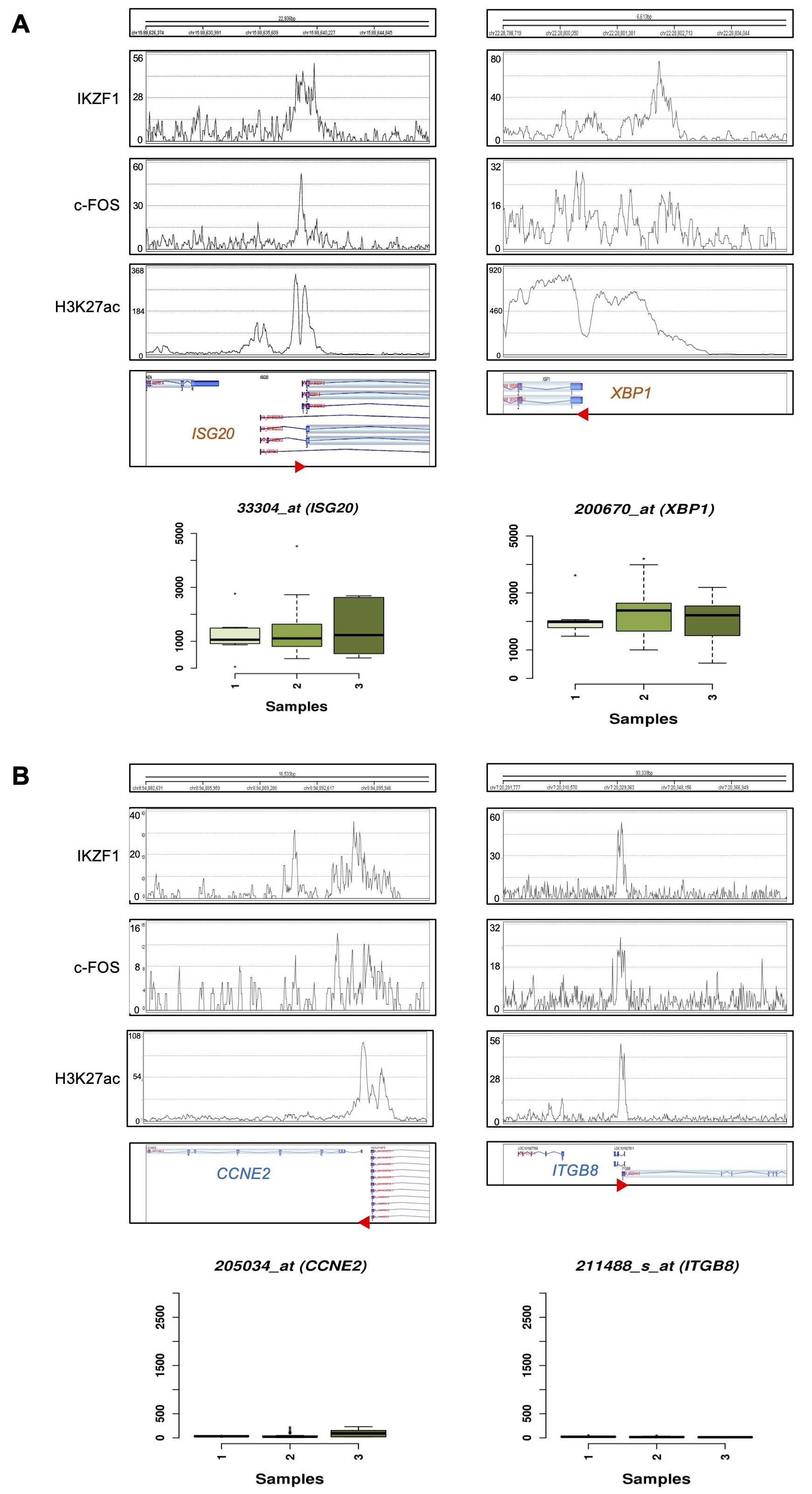


**Supplemental Figure 3. Co-occupancy of IKZF1 and c-FOS at promoter/enhancer regions of actively transcribed genes in MM.** Upper panel: ChIP-seq data of IKZF1 and c-FOS binding in MM.1S cells aligned with histone H3K27 acetylation in the UCSC genome browser. The TSS of each gene is shown by red triangles. Lower panel: Gene expression was assayed using Affymetrix U133 plus 2.0 microarrays. Data are unpaired GCRMA-normalized expression signals for each gene in CD138-positive cells from patients with (1) monoclonal gammopathy of undetermined significance, (2) MM, and (3) plasma cell leukemia (n=8 each) [53]. **(A)** The results of two representative genes that are highly expressed in plasma cell disorders. **(B)** The results of two representative genes not expressed in plasma cell disorders.


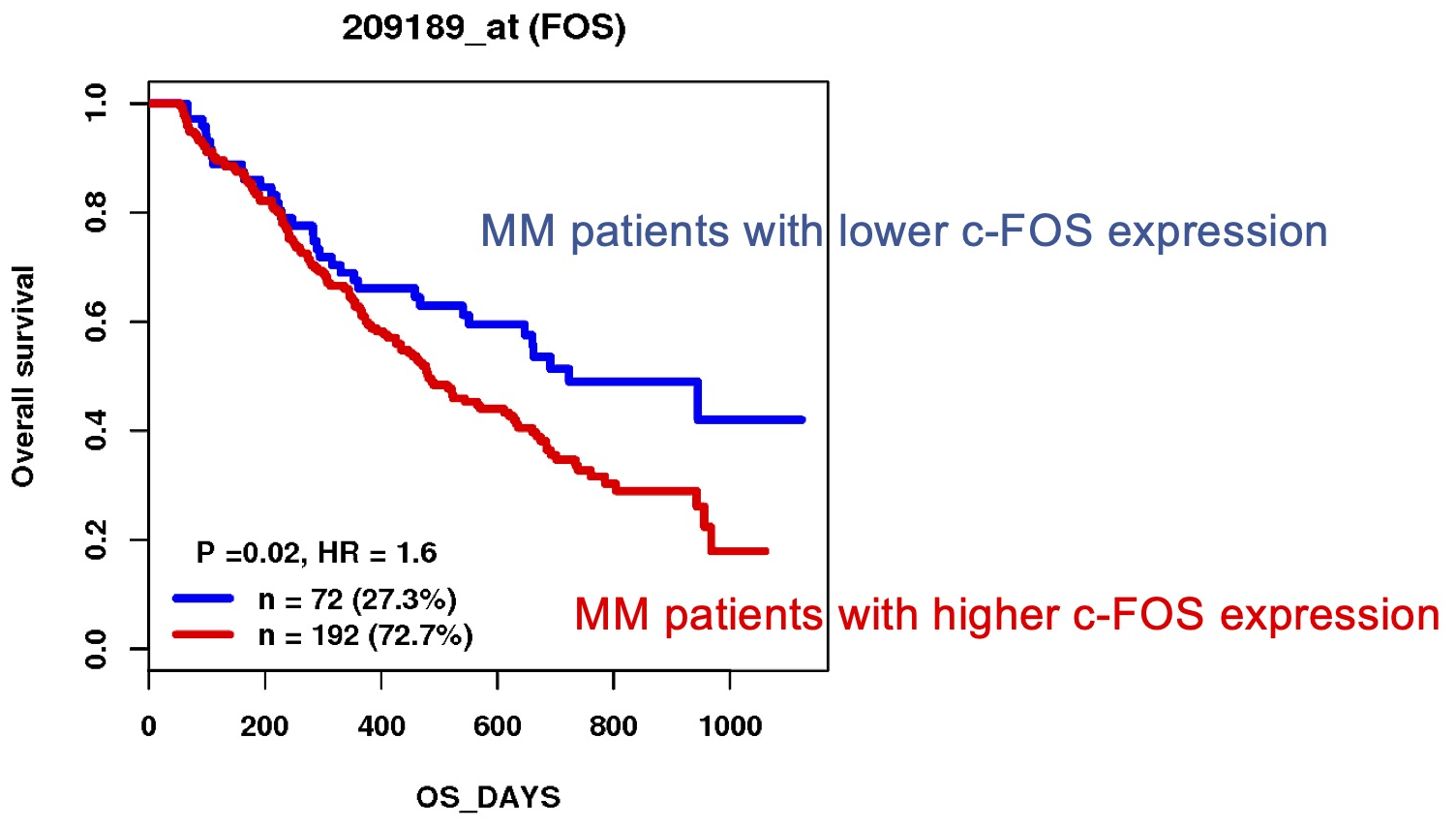


**Supplemental Figure 4. The effects of the expression levels of c-FOS on the survival of MM patients.** We generated the Kaplan-Meier survival curves of 264 newly-diagnosed MM patients treated with Total Therapy 2/3 using the data deposited in the GenomicScape database [53]. Red: MM patients whose tumor cells expressed *FOS* mRNA higher than normal plasma cells. Blue: MM patients whose tumor cells expressed *FOS* mRNA lower than normal plasma cells. *P*=0.02 by the log-rank test.


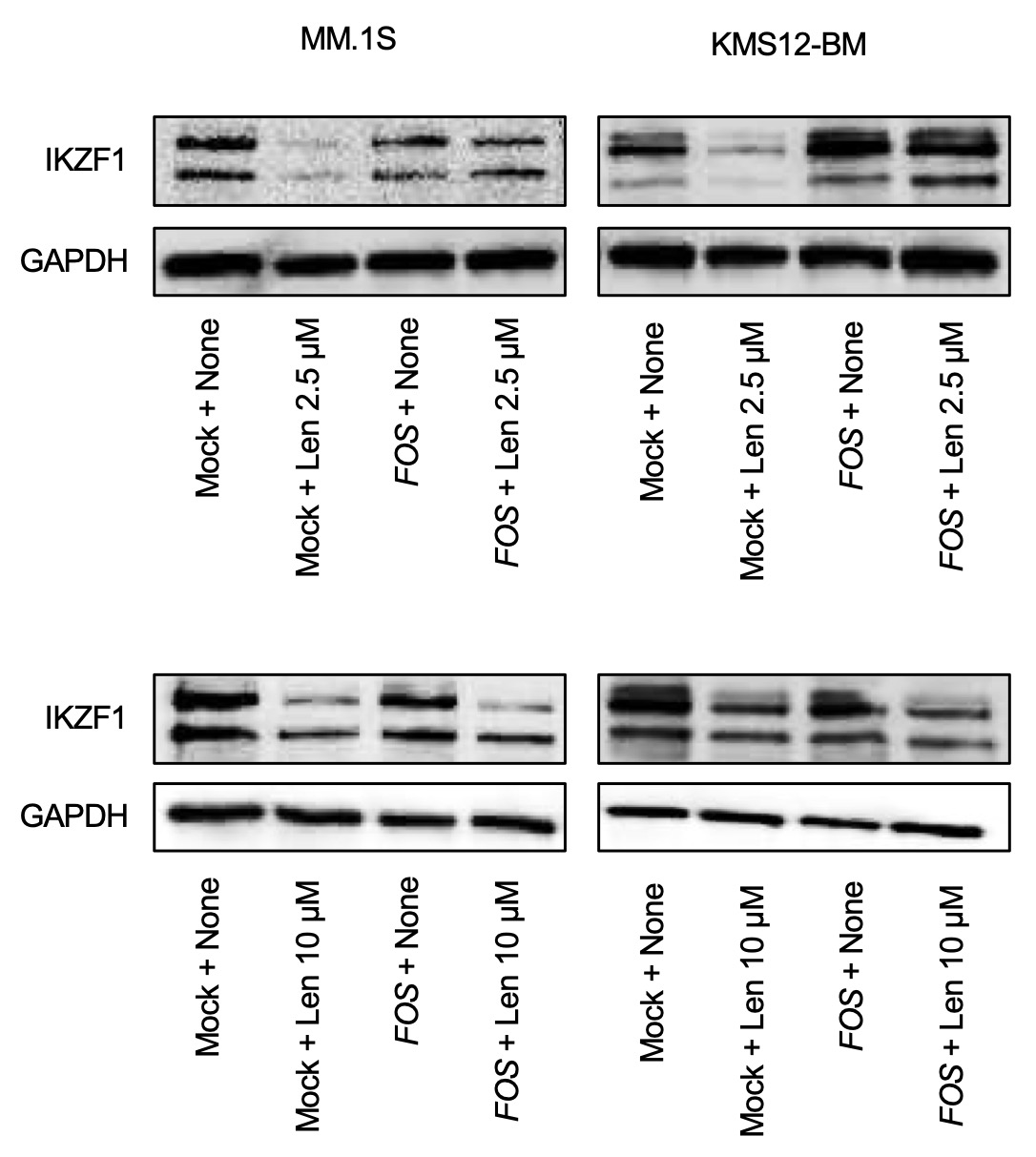


**Supplemental Figure 5. The expression of IKZF1 in c-FOS-overexpressing MM cell lines with or without lenalidomide treatment.** MM.1S and KMS12-BM cells were transfected with *FOS* expression vector or empty vector (Mock) and then treated with the vehicle alone (None) or lenalidomide (2.5 μM or 10 μM) for 24 hours, followed by immunoblot analysis for the expression of IKZF1 and GAPDH (loading control).


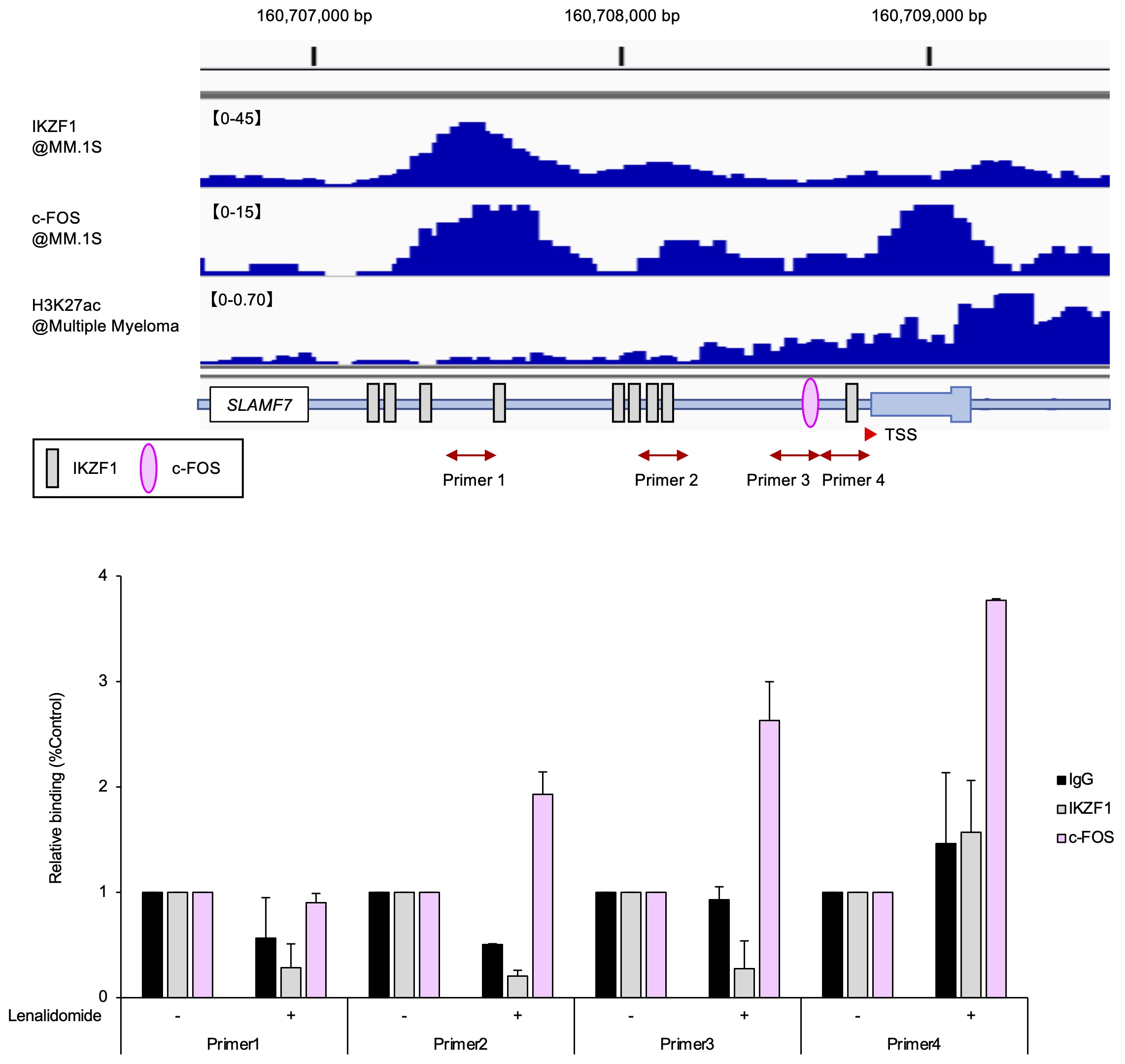


**Supplemental Figure 6. Effects of lenalidomide on IKZF1 and c-FOS binding at promoter/enhancer regions of the *SLAMF7* gene in MM cells.** Upper panel: Schematic representation of the *SLAMF7* promoter region from the ChIP-Atlas data. The relative positions of the putative binding sites of transcription factors are approximated by the symbols shown in the box. TSS; transcription start site. Bidirectional red arrows indicate regions that were PCR amplified in ChIP assays. Lower panel: Chromatin suspensions were prepared from KMS12-BM cells cultured with vehicle alone or 2.5 μM lenalidomide for 24 hours and immunoprecipitated with anti-IKZF1 (gray bars) and c-FOS (pink bars) antibodies or IgG (back bars). The resulting precipitates were subjected to PCR to amplify the regions shown in the upper panel.


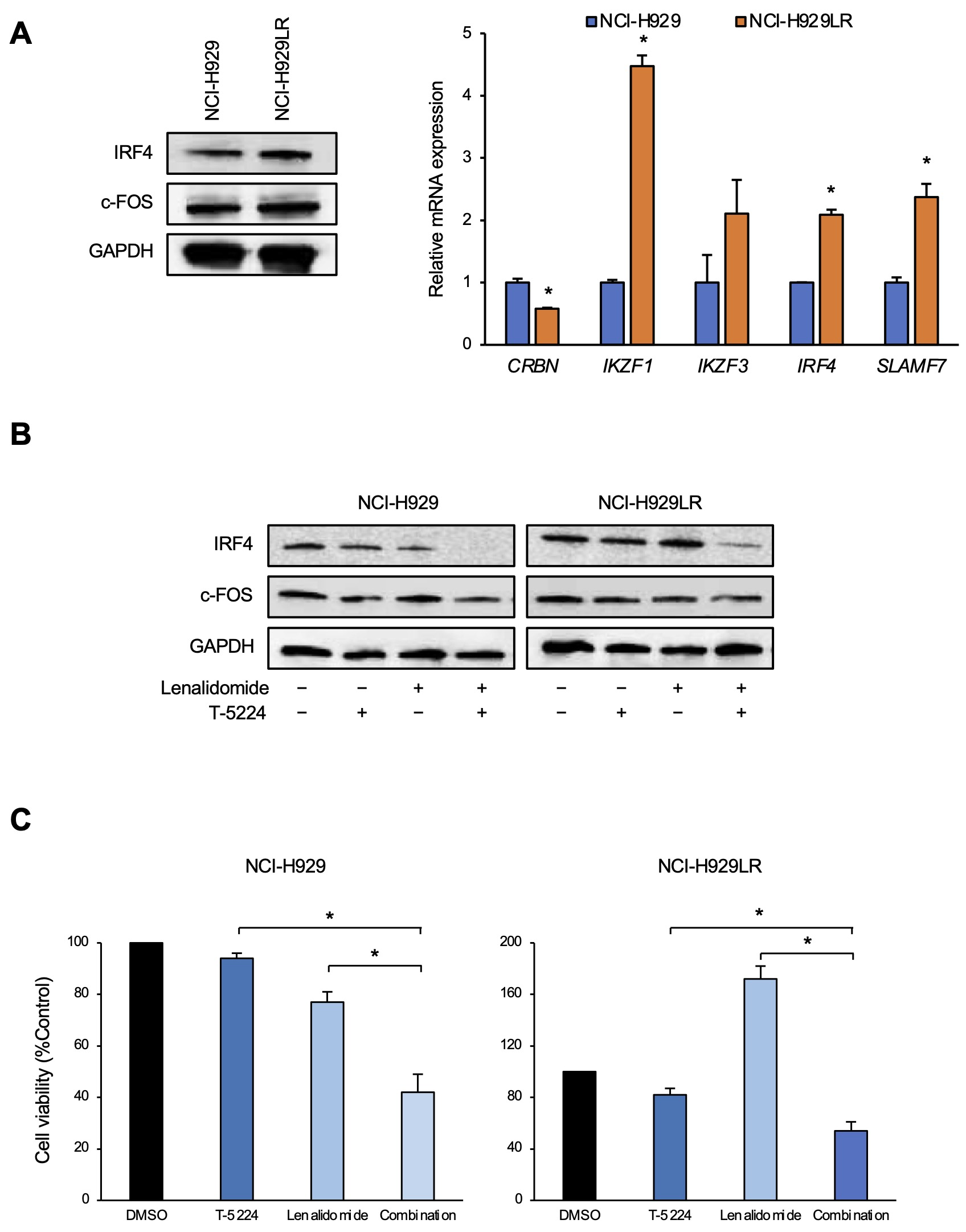


**Supplemental Figure 7. A selective AP-1 inhibitor overcomes lenalidomide resistance of NCI-H929 cells. (A)** We established lenalidomide-resistant sublines of the NCI-H929 cell line via long-term exposure to lenalidomide and designated them NCI-H929LR. Left panel: We examined the expression of IRF4, c-FOS and GAPDH (loading control) in NCI-H929LR and parental NCI-H929 cells using immunoblotting. Right panel: We examined the expression of the indicated genes in NCI-H929LR and parental NCI-H929 cells using quantitative real-time reverse transcription-PCR. **P* <0.05 by Student’s *t*-test (n=3). **(B)** We treated NCI-H929LR and parental NCI-H929 cells with the indicated combination of lenalidomide (10 μM) and the selective AP-1 inhibitor T-5224 (20 μM) for 24 hours and examined the expression of the indicated molecules using immunoblotting. **(C)** We cultured NCI-H929LR and parental NCI-H929 cells with 0.1% DMSO (vehicle), lenalidomide (10 μM), T-5224 (20 μM) or the combination of lenalidomide and T-5224 for 48 hours and examined the cell viability using the MTT reduction assay. **P* <0.05 by Student’s *t*-test (n=3).


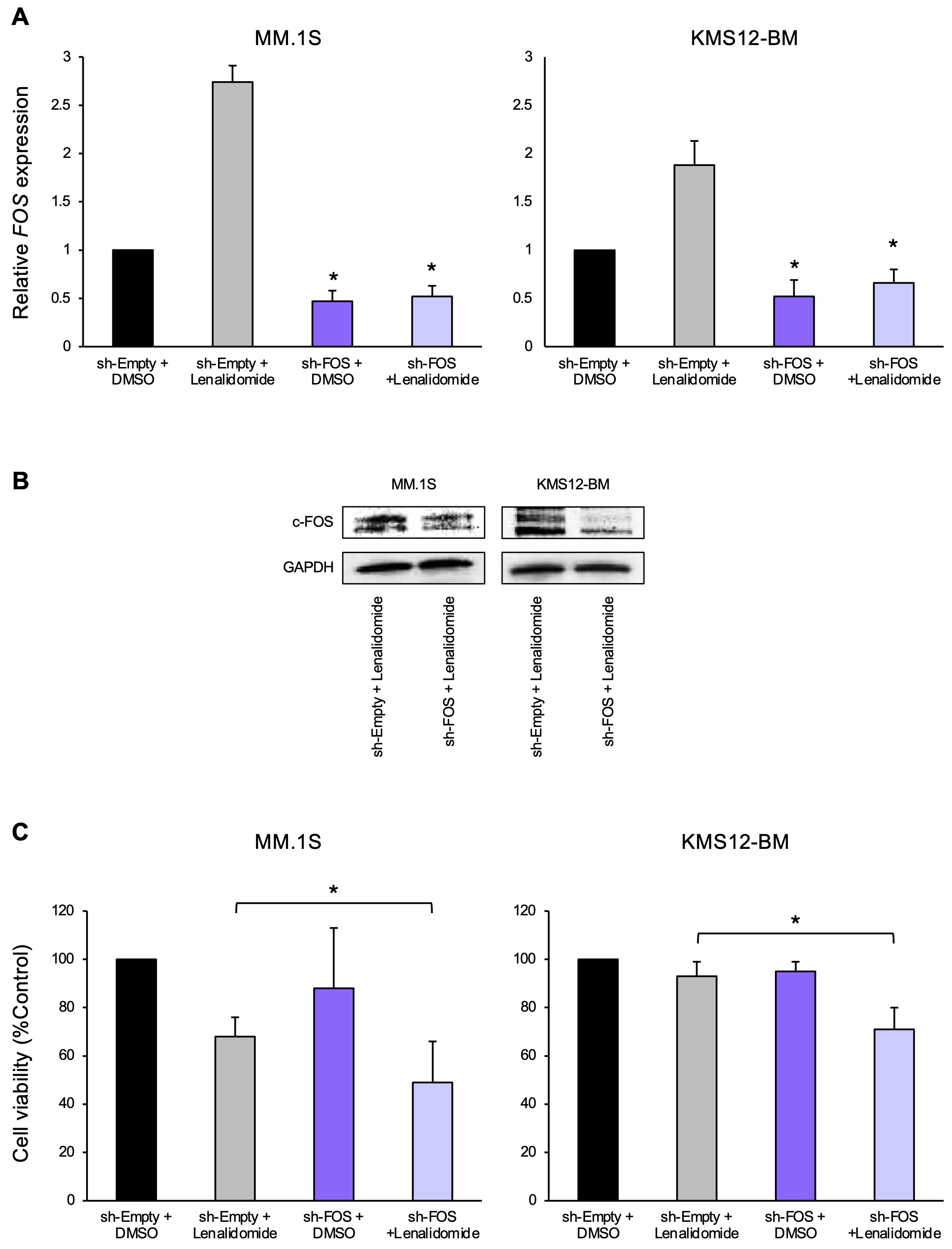


**Supplemental Figure 8. The effects of c-FOS knockdown on MM cells. (A)** We transduced MM.1S and KMS12-BM cells with the vector carrying sh-RNA against *FOS* (sh-FOS) or an empty vector (sh-Empty) and treated them with vehicle alone (0.1% DMSO) or 10 μM lenalidomide for 24 hours, followed by quantitative real-time reverse transcription-PCR for the expression of *FOS* and *GAPDH* transcripts. Data were quantified by the 2^–∆∆Ct^ method using *GAPDH* as a reference and are shown as the fold changes against the values of DMSO-treated sh-Empty cells. **P* <0.05 by one-way ANOVA with Student–Newman–Keuls multiple comparison test. **(B)** Whole cell lysates were isolated from the indicated cells and subjected to immunoblot analyses for the expression of c-FOS and GAPDH (loading control). **(C)** Cell viability was determined using the MTT reduction assay after 72 hours of the culture described in **(A)**. **P* <0.05 by one-way ANOVA with Student–Newman–Keuls multiple comparison test.


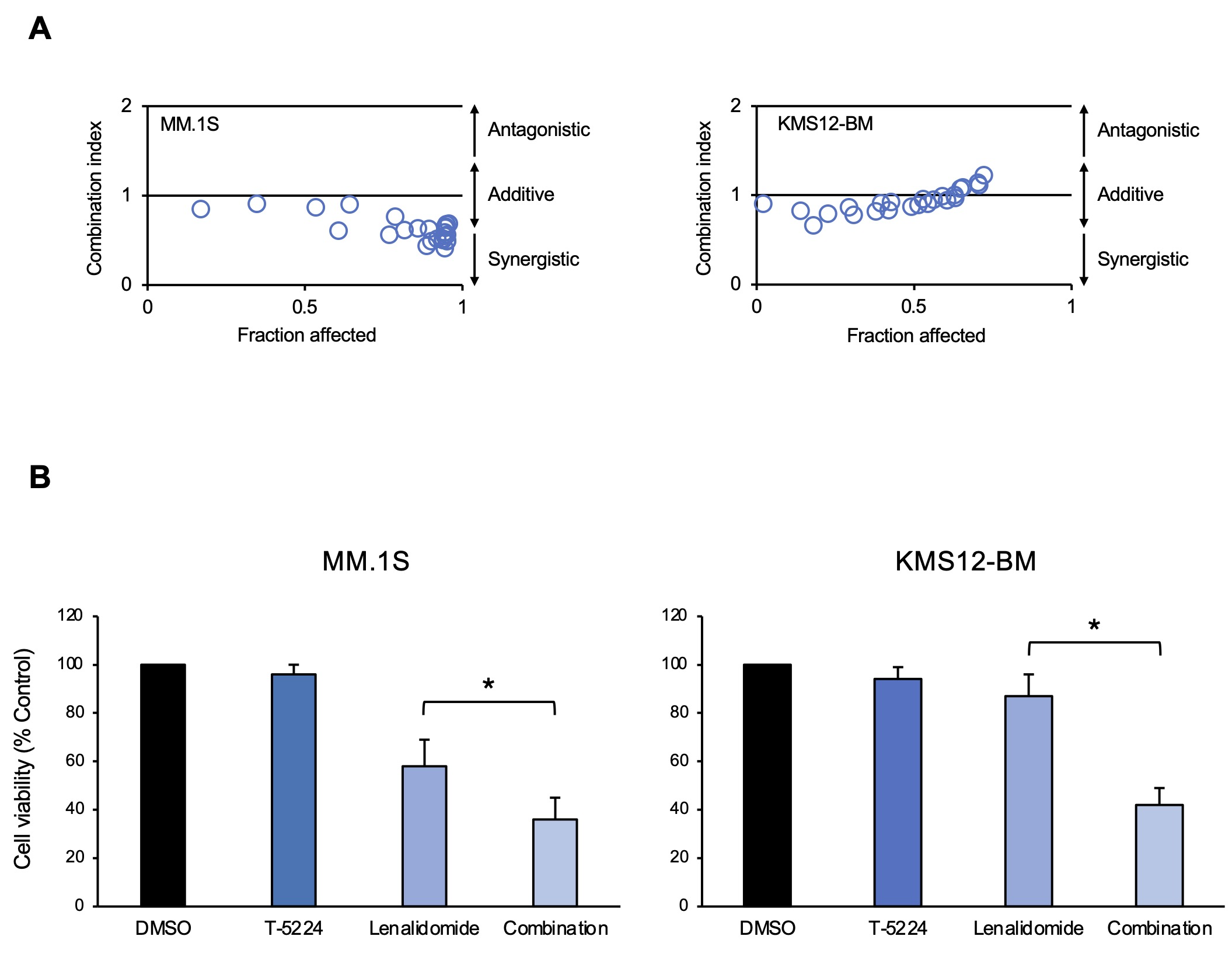


**Supplemental Figure 9. The combined effects of a selective AP-1 inhibitor and lenalidomide in MM cells.**

**(A)** MM.1S and KMS12-BM cells were treated with lenalidomide in combination with T-5224 in 96-well plates for 72 hours. Dose-response curves of each combination were generated to construct nonconstant normalized isobolograms at IC_50_ using CompuSyn software (http://www.combosyn.com). The isobolograms shown are representative of at least three independent experiments. Combination indexes <0.8 and 0.8-1.2 indicate the synergism and additivity between the two drugs, respectively. **(B)** MM.1S and KMS12-BM cells were cultured with 0.1% DMSO (vehicle), lenalidomide (10 μM), T-5224 (20 μM) or the combination of lenalidomide and T-5224 for 72 hours. Cell viability was determined by the MTT reduction assay. **P* <0.05 by Student’s *t*-test (n=3).


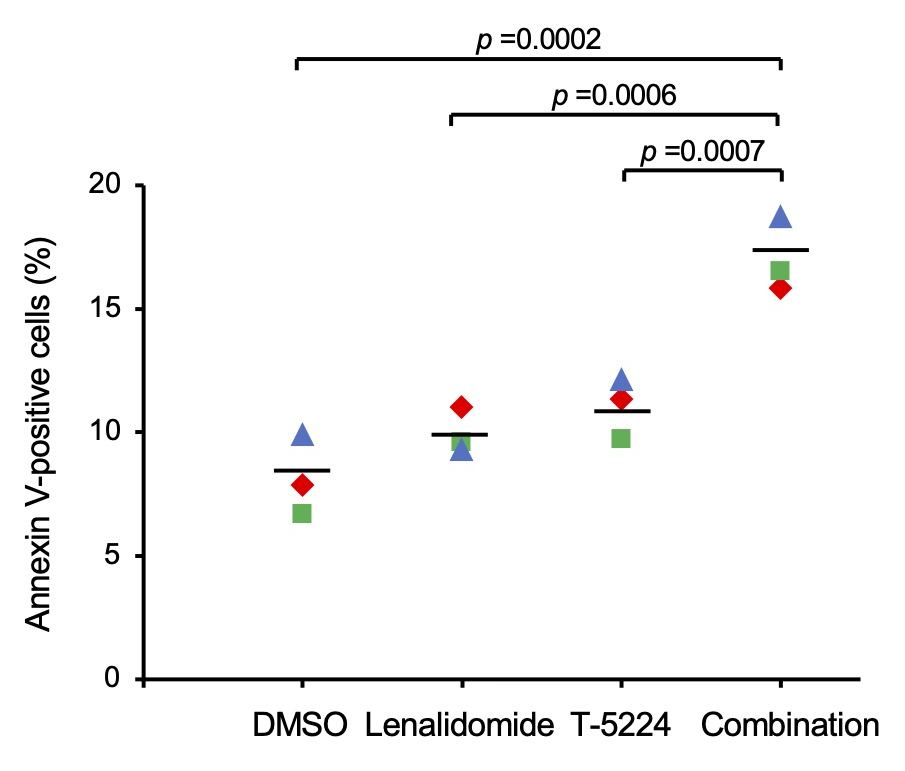


**Supplemental Figure 10. The combined effects of a selective AP-1 inhibitor and lenalidomide in primary MM cells.** CD138-positive cells were isolated from the bone marrow of three newly-diagnosed MM patients and cultured with 0.1% DMSO (vehicle), lenalidomide (10 μM), T-5224 (20 μM) or the combination of lenalidomide and T-5224 for 72 hours. Cell viability was determined by flow cytometric analysis of annexin V positivity. **P* <0.05 by one-way ANOVA with Student–Newman–Keuls multiple comparison test.


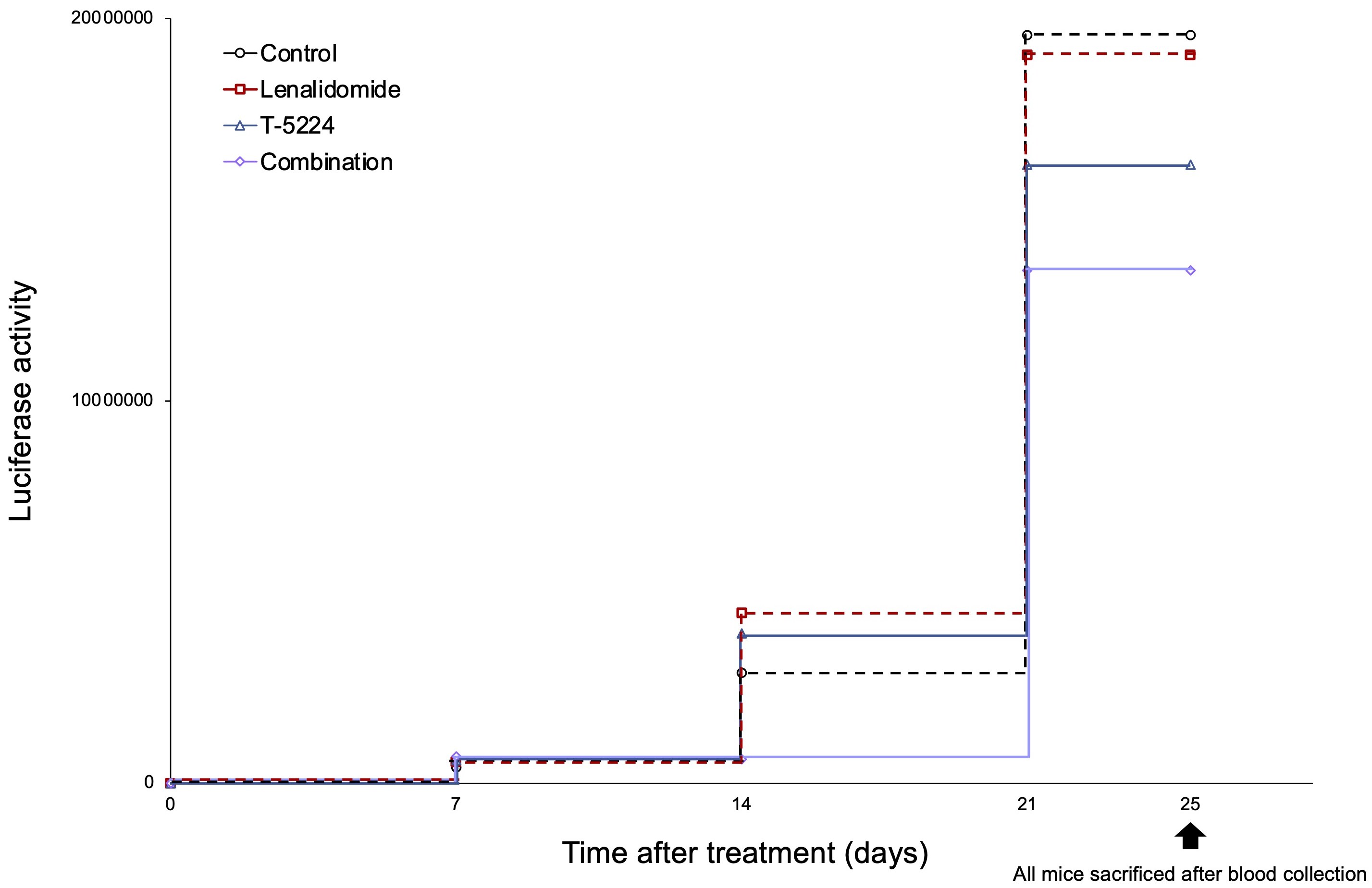


**Supplemental Figure 11. A selective AP-1 inhibitor augments the effects of lenalidomide in a murine MM model.** Luciferase-expressing MM.1S cells were inoculated subcutaneously into the right thigh of NOD/SCID mice (1 × 10^6^ cells/mouse). When the inoculated tumor became measurable (defined as day 1), we started treatment with vehicle alone (0.9% NaCl, twice a week), 20 mg/kg T-5224 (twice a week), 10 mg/kg lenalidomide (twice a week), or the combination of T-5224 and lenalidomide (20 mg/kg T-5224 and 10 mg/kg lenalidomide, twice a week) intraperitoneally for two weeks. The tumor burden was monitored by measuring tumor-derived luciferase activity with a noninvasive bioimaging system on the indicated days. In short, tumor-bearing mice were intraperitoneally injected with 1.5 mg of the luciferase substrate *D*-luciferin after being anesthetized with isoflurane. Photons transmitted through the body were collected for a specified length of time and analyzed using the IVIS-CT Imaging System with Living Image software (Xenogen, Alameda, CA). Quantitative data were expressed as photon units (photons/s).

Supplemental Table 1. Oligonucleotide sequences used for knockdown experiments

--------------------------------------------------------------------------------------------------------------------------------------------

Gene Sequence (sequences corresponding to *FOS* are in lowercase)

--------------------------------------------------------------------------------------------------------------------------------------------*FOS* Forward: 5'-TgaccaatattatactaagaTTCAAGAGAtcttagtataatattggtcTTTTTTC-3'

Reverse: 5'-TCGAGAAAAAAgaccaatattatactaagaTCTCTTGAAtcttagtataatattggtcA-3'

--------------------------------------------------------------------------------------------------------------------------------------------

Supplemental Table 2. Oligonucleotide sequences used for ChIP assays

--------------------------------------------------------------------------------------------------------------------------------------------

Gene Sequence (corresponding nucleotide positions) Product size

--------------------------------------------------------------------------------------------------------------------------------------------

Primer 1 for *IRF4* Forward: 5'-CAGCATCTCAGACGATCGAA-3' (–526 to –506) 103 bp Reverse: 5'-TTAAGTGGACCAGGGATTTTACA-3' (–446 to –423)

Primer 2 for *IRF4* Forward: 5'-GTTGGCCAGGCTGGTCTC-3' (–239 to –221) 150 bp

Reverse: 5'-AACCTCAACCTGGAGGAAAGC-3' (–110 to –89)

Primer 3 for *IRF4* Forward: 5'-TCACTTCAATTCACCAGCCTAA-3' (+43 to +65) 100 bp

Reverse: 5'-CCCAGCTCCCTTGAGCTATT-3' (+123 to +143)

Primer 4 for *IRF4* Forward: 5'-GCTCTGCAAAGCGAAGTCC-3' (+287 to +306) 109 bp

Reverse: 5'-ACCTCAGGAGGCCAGTCAAT-3' (+376 to +396)

Primer 1 for *SLAMF7* Forward: 5'-ACACAGGATGGCCAGGAA-3' (–950 to –932) 100 bp

Reverse: 5'-TCAGAGCTTAAGTTTGCCATGT-3' (–872 to –850)

Primer 2 for *SLAMF7* Forward: 5'-GATGGCTGATTTCTCACTGG-3' (–566 to –546) 113 bp

Reverse: 5'-TTCATTGAGAACCATATCAGGTAA-3' (–477 to –453)

Primer 3 for *SLAMF7* Forward: 5'-GCCAAAGACCCCGTTAAGA-3' (–273 to –254) 113 bp

Reverse: 5'-TGACAATTCCTTATATGTTCCAAGT-3' (–185 to –160)

Primer 4 for *SLAMF7* Forward: 5'-TTCACCAAGGAGGATATGGA-3' (–96 to –76) 97 bp

Reverse: 5'-TCATTGTGGCTTTAATTTGCAT-3' (–18 to +1)

--------------------------------------------------------------------------------------------------------------------------------------------
